# TGF-**β** signaling promotes astroglial activation and TDP-43 proteinopathy in organoid models of frontotemporal lobar degeneration

**DOI:** 10.1172/JCI190035

**Published:** 2026-06-16

**Authors:** Arren C. Ramsey, Xiao-Yan Tang, Magdalena J. Macias, Patricia R. Nano, Rufei Lu, Brian Benito, Cameron M. Lau, Jisu Park, Jiasheng Zhang, Wandy Beatty, Tanzila Mukhtar, Arnold R. Kriegstein, Aparna Bhaduri, Elise Marsan, Eric J. Huang

**Affiliations:** 1 Department of Pathology,; 2 BMS Graduate Program, and; 3 Weill Institute for Neurosciences, UCSF, San Francisco, California, USA.; 4 Department of Biological Chemistry, UCLA, Los Angeles, California, USA.; 5 Department of Pathology & Immunology and; 6 Department of Molecular Microbiology, Washington University School of Medicine, St. Louis, Missouri, USA.; 7 The Eli and Edythe Broad Center of Regeneration Medicine and Stem Cell Research and; 8 Department of Neurology, UCSF, San Francisco, California, USA.; 9 Center for Alzheimer’s and Related Dementias, NIH, Bethesda, Maryland, USA.

**Keywords:** Aging, Neuroscience, Dementia, Neurodegeneration, iPS cells

## Abstract

Dominant mutations in progranulin (*GRN*) gene cause frontotemporal lobar degeneration (FTLD-GRN), whereas homozygous *GRN* mutations lead to neuronal ceroid lipofuscinosis, a childhood neurodegenerative disorder. While recent transcriptomic studies reveal profound glial and neuronal pathology in FTLD-GRN at the disease end stage, the mechanism that disrupts glia-neuron homeostasis remains unclear. Using induced pluripotent stem cell–derived cortical organoids, we showed that *GRN^–/–^* and *GRN^R493X^* mutations led to precocious astrogliosis that promoted neuronal stress and synaptic loss. Single-cell transcriptomics and histopathology analyses revealed a robust activation in the TGF-β signaling pathway in *GRN^–/–^* and *GRN^R493X/R493X^* astrocytes, which was accompanied by features of immune activation, loss of synaptic support, and abundant pTDP-43^+^ fibrils in astroglial cytoplasm, a feature characteristic of FTLD-GRN. Intriguingly, blocking TGF-β signaling mitigated astroglial activation and pTDP-43 proteinopathy in *GRN^–/–^* organoids. Together, these results provide insights into the cell-autonomous role of astroglial activation in neurodegeneration caused by progranulin deficiency.

## Abstract 2

Rasmey et al. used induced pluripotent stem cell (iPSC)-derived cortical organoids to show that homozygous loss-of-function or dominant mutations in *GRN* lead to precocious astrogliogenesis and aberrant astroglial activation. Furthermore, single-cell transcriptomics identified aberrant activation of TGFb signaling in promoting immune activation and TDP-43 proteinopathy in *GRN^-/-^* and *GRN^R493X/R493X^* astrocytes.

## Graphical abstract

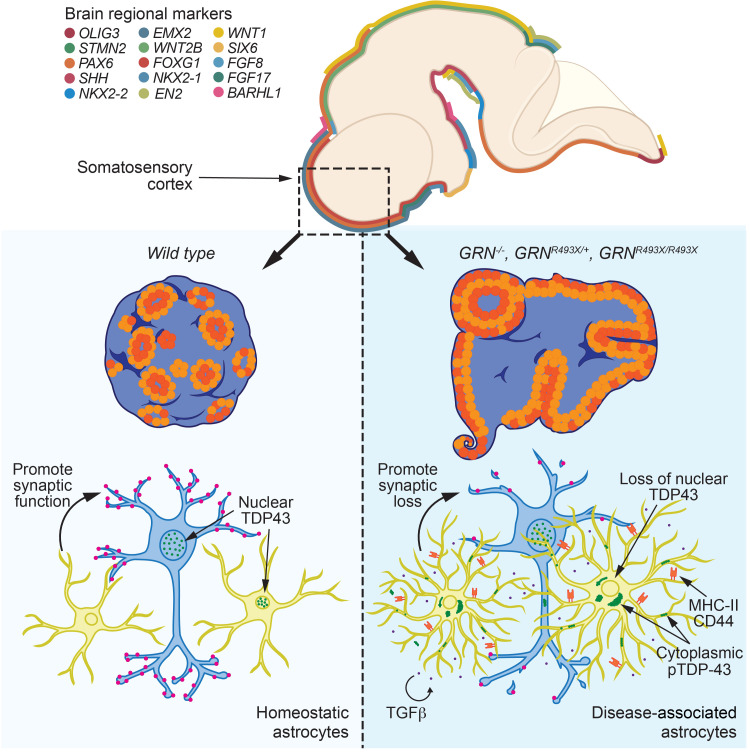

## Introduction

Haploinsufficiency in human progranulin (*GRN*) gene causes frontotemporal lobar degeneration (FTLD), a common neurodegenerative disease affecting patients under 60 (1, 2). In addition, homozygous mutations in *GRN* are the genetic causes for neuronal ceroid lipofuscinosis (CLN11) (3–5), which lead to neurodegenerative disease starting in childhood. Although the timing of disease onset is different, FTLD-GRN and CLN11 share several distinct neuropathological features, including the prominent accumulation of lysosomal storage materials, such as gangliosides, in brain tissues (6). The mechanism responsible for the accumulation of lysosomal storage materials in FTLD-GRN and CLN11 is due to the essential role of *GRN* gene product progranulin (PGRN) in regulating the transition of late endosomes to early lysosomes (7). Indeed, PGRN is known to interact with several lysosomal proteins, including cathepsin D, prosaposin, and TMEM106B, which when deleted leads to similar lysosomal defects (8–12).

One key neuropathological feature of FTLD-GRN is the presence of TDP-43 proteinopathy in the frontal cortex and thalamus, which is characterized by the accumulation of RNA binding protein TDP-43 in neuronal cytoplasm and in many thread-like inclusions in unknown cell types (2, 13). Using single-cell transcriptomics, we and others have uncovered highly conserved transcriptomic signatures in astrocytes in the frontal cortex, temporal cortex, and thalamus of patients with FTLD-GRN, which suggest that aberrant activation of these astrocytes may contribute to neuronal degeneration (14, 15). In support of this idea, induced pluripotent stem cell–derived (iPSC-derived) *GRN^–/–^* astrocytes promote synaptic degeneration and neuronal stress when transplanted onto cortical organoids (15). However, it remains unclear how PGRN-deficient astrocytes contribute to TDP-43 proteinopathy.

Using iPSC-derived cortical organoids as a model system, we show that loss of function in *GRN* and dominant *GRN^R493X^* mutation lead to neurodevelopmental defects and precocious astrogliosis. Single-cell RNA-sequencing (scRNA-seq) and histopathology analyses reveal that *GRN^–/–^* astrocytes exhibit a robust activation in TGF-β signaling pathways and features of immune activation, loss of synaptic support, and pTDP-43^+^ fibrils in astroglial cytoplasm. Interestingly, blocking TGF-β signaling mitigates key aberrant features in *GRN^–/–^* astrocytes, including the formation of pTDP-43^+^ fibrils. These results provide key mechanistic insights into the cell-autonomous role of astroglial activation caused by PGRN deficiency.

## Results

### Loss of PGRN leads to aberrant growth in cortical organoids.

**Figure 1:**
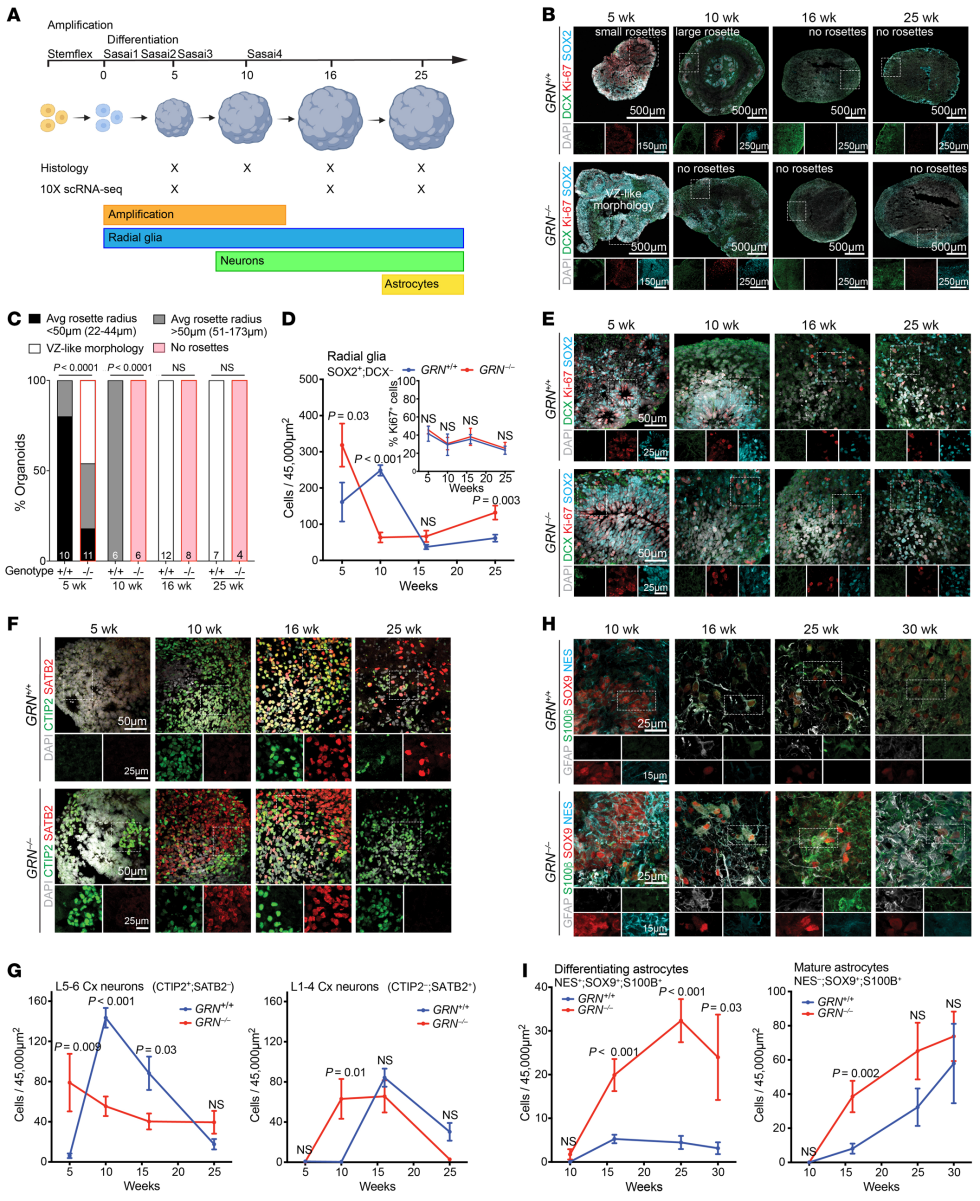
Modeling progranulin deficiency in neurodevelopment using iPSC-derived cortical organoids. (**A**) Schematic representation of the generation and analysis of iPSC-derived *GRN^+/+^* and *GRN^–/–^* cortical organoids. (**B**) Confocal images of DCX, Ki-67, and SOX2 in *GRN^+/+^* and *GRN^–/–^* cortical organoids at 5, 10, 16, and 25 weeks. DCX, doublecortin. (**C**) Percentage of organoids exhibiting different rosette morphologies at various developmental time points in *GRN^+/+^* and *GRN^–/–^* cases. Rosette morphologies are categorized by average rosette radius: <50 μm (black), >50 μm (gray), VZ-like morphology (white), and no rosettes (striped). Organoids (*n* = 9) from 3 independent biological replicate experiments were analyzed per time point for each genotype. VZ, ventricular zone. (**D**) Quantification of the density of SOX2^+^DCX^–^ radial glia cells at different time points in *GRN^+/+^* (blue) and *GRN^–/–^* (red) groups. Inset shows the percentage of Ki-67^+^ cells among SOX2^+^DCX^–^ radial glia over time, with no significant differences (NS) observed between groups. Organoids (*n* = 9) from 3 independent biological replicate experiments were analyzed per time point per genotype. (**E**) Confocal images of DCX, Ki-67, and SOX2 in *GRN^–/–^* and *GRN^+/+^* iPSC-derived cortical organoids at different developmental time points. (**F**) Confocal images of CTIP2, SATB2 in *GRN^–/–^* and *GRN^+/+^* iPSC-derived cortical organoids at different time points. (**G**) Percentage of CTIP2^+^SATB2^–^ deep-layer neurons and CTIP2^–^SATB2^+^ upper-layer neurons at different time points in *GRN^+/+^* (blue) and *GRN^–/–^* (red) groups. Organoids (*n* = 9) from 3 independent biological replicate experiments were analyzed per time point for each genotype. (**H**) Confocal images of glial fibrillary acidic protein (GFAP), S100β, SOX9, and NESTIN (NES) in *GRN^–/–^* and *GRN^+/+^* iPSC-derived cortical organoids at different time points. (**I**) Percentage of NES^+^SOX9^–^S100β^+^ differentiating astrocytes and NES^–^SOX9^+^S100β^+^ mature astrocytes at different time points in *GRN^+/+^* (blue) and *GRN^–/–^* (red) groups. Organoids (*n* = 9) from 3 independent biological replicates were analyzed per time point per genotype. All data represent mean ± SEM.

To determine how PGRN deficiency might impact neurodevelopment, we differentiated wild-type (*GRN^+/+^*) WTC11 and isogenic *GRN^–/–^* iPSCs into cortical organoids using protocols previously established (15–17) (Figure 1A). Using morphological features and cell type–specific markers, we showed that *GRN^+/+^* cortical organoids (organoids from here onward) developed relatively uniform rosettes (22–44 μm in diameter), which contained SOX2^+^Ki-67^+^ neural progenitors at 5 weeks. These rosettes grew bigger at 10 weeks but became undetectable at 16 and 25 weeks (Figure 1B and Supplemental Figure 1A; supplemental material available online with this article; https://doi.org/10.1172/JCI190035DS1). In contrast, *GRN^–/–^* organoids were larger than *GRN^+/+^* organoids at 5 weeks and developed fewer but larger rosettes (51–173 μm in diameter) that can be detected as early as 4 weeks (Figure 1, B and C, and Supplemental Figure 1B). About 50% of *GRN^–/–^* organoids at this early age showed VZ-like morphology. Interestingly, from 10 to 25 weeks, no rosettes were identified in *GRN^–/–^* organoids (Figure 1, B and C). To further examine how PGRN deficiency impacts neurogenesis, we stained *GRN^+/+^* and *GRN^–/–^* organoids for SOX2, Ki-67, and neuroblast marker DCX. In *GRN^+/+^* organoids, the number of SOX2^+^DCX^–^ neural progenitors expanded from 5 to 10 weeks followed by a significant reduction at 16 and 25 weeks. In contrast, *GRN^–/–^* organoids exhibited an early expansion of SOX2^+^DCX^–^ progenitors at 5 weeks before dropping down at 10 weeks (Figure 1, D and E). By 25 weeks, *GRN^–/–^* organoids showed significantly more SOX2^+^DCX^–^ progenitors. In addition to the early expansion of SOX2^+^DCX^–^ progenitors, *GRN^–/–^* organoids also had a modest increase in SOX2^+^DCX^+^ transitioning neuroblasts at 10 weeks and no significant increase in SOX2^–^DCX^+^ young neuroblasts at all stages (Supplemental Figure 1C). Despite the more robust growth in *GRN^–/–^* organoids, no difference was observed in the proportion of Ki-67^+^ cells in SOX2^+^DCX^–^ progenitors, SOX2^+^DCX^+^ transitioning neuroblasts, or SOX2^–^DCX^+^ young neuroblasts between *GRN^+/+^* and *GRN^–/–^* organoids (Figure 1D and Supplemental Figure 1C).

Given the early expansion of SOX2^+^DCX^–^ progenitors in *GRN^–/–^* organoids, we asked if loss of PGRN might lead to precocious neurogenesis. To investigate this, we stained *GRN^+/+^* and *GRN^–/–^* organoids for layer-specific cortical neuron marker SATB2 (layers 1–4 or L1–4) and CTIP2 (L5–6). In *GRN^+/+^* organoids, CTIP2^+^SATB2^–^ neurons exhibited a significant expansion at 10 weeks, followed by a progressive decrease at 16 and 25 weeks, whereas CTIP2^–^SATB2^+^ neurons were detected at 16 weeks followed by a decrease at 25 weeks (Figure 1, F and G). In *GRN^–/–^* organoids, however, a significant number of CTIP2^+^ SATB2^–^ neurons could be detected as early as 4 weeks (Supplemental Figure 1D), and these neurons persisted at 10, 16, and 25 weeks. Similarly, *GRN^–/–^* organoids showed precocious differentiation of CTIP2^–^SATB2^+^ neurons at 10 weeks. These neurons persisted at 16 weeks and disappeared at 25 weeks (Figure 1, F and G). Interestingly, *GRN^+/+^* organoids contained a small number of transient CTIP2^+^SATB2^+^ neuron population at 16 weeks; this neuronal population was nearly undetected in *GRN^–/–^* organoids at all stages (Supplemental Figure 1E).

Next, we used astrocyte markers GFAP, S100B, and SOX9 to capture different stages of astroglial differentiation and determine how PGRN deficiency impacted this process. In addition, we included radial glia marker NESTIN (NES) to distinguish radial glia from astrocytes (Figure 1H). Recognizing that astroglial differentiation likely occurs much later than neurogenesis, we extended our analyses on *GRN^+/+^* and *GRN^–/–^* organoids to 30 weeks. Compared with *GRN^+/+^* organoids, *GRN^–/–^* organoids exhibited no difference in the number of astroglial progenitor populations (NES^+^SOX9^+^S100B^–^ and NES^–^SOX9^+^S100B^–^) (Supplemental Figure 1F). Rather, *GRN^–/–^* organoids consistently contained more differentiating astrocytes (NES^+^SOX9^+^S100B^+^) at 16, 25, and 30 weeks and mature astrocytes (NES^–^SOX9^+^S100B^+^) at 16 weeks (Figure 1, H and I). The percentage of GFAP^+^ astrocytes in each of these subgroups did not differ between genotypes (Supplemental Figure 1G). One notable feature of *GRN^–/–^* astrocytes was bulky soma compared with *GRN^+/+^* astrocytes at all ages (Figure 1H).

### Precocious neurogenesis and astrogliogenesis in GRN^–/–^ cortical organoids.

**Figure 2:**
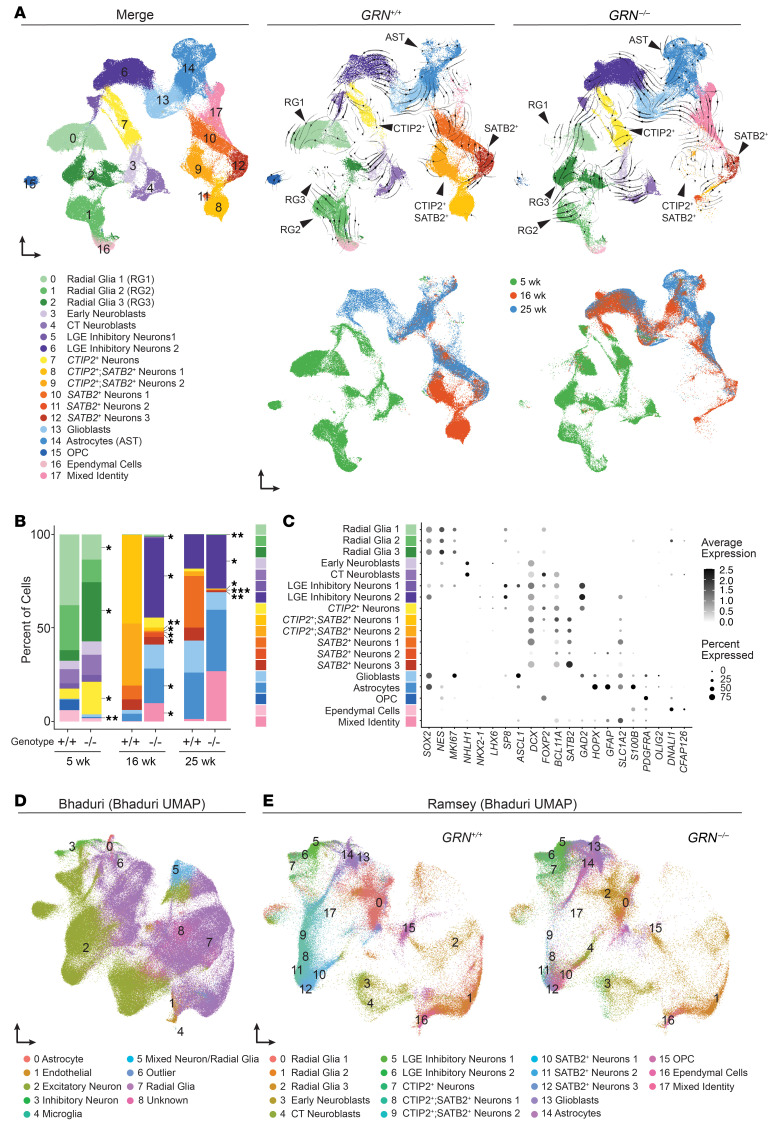
Single-cell transcriptomics of *GRN^+/+^* and *GRN^–/–^* cortical organoids. (**A**) Uniform manifold approximation and projections (UMAPs) of combined (left), *GRN^+/+^* (center), and *GRN^–/–^* (right) cortical organoids grouped by cluster with an overlay of RNA velocity vectors (top row) and UMAPs of *GRN^+/+^* (center) and *GRN^–/–^* (right) cortical organoids grouped by age (bottom row). (**B**) Bar graphs of the percentage of cells in each cluster split by age and genotype. (*P ≤ 0.05, **P ≤ 0.01, ***P ≤ 0.001.) Statistics used unpaired 2-tailed *t*-test for normally distributed data and Mann-Whitney U test for not normally distributed data. (**C**) Dot plots of representative genes used to identify each cluster. (**D** and **E**) UMAPs comparing the scRNA-seq dataset from Bhaduri et al. (16) grouped by Bhaduri clusters (**D**) and the scRNA-seq dataset from *GRN^+/+^* and *GRN^–/–^* cortical organoid datasets (this study) projected onto the Bhaduri UMAP clusters (**E**).

To provide further insights into how PGRN deficiency impacts the developmental trajectories of different cell types in organoids, we conducted scRNA-seq on *GRN^+/+^* and *GRN^–/–^* organoids at 5, 16, and 25 weeks (Figure 1A). In total, we used 4–6 biological replicates and 2 experimental replicates for each time point and genotype. After quality control, we obtained 29,757–54,465 cells per time point/genotype, with 8,930 reads and 3,180 genes per cell (Supplemental Figure 2, A–D). Unsupervised clustering resulted in 18 clusters, including 3 clusters of radial glia, early neuroblasts, cortico-thalamic (CT) neuroblasts, 2 clusters of inhibitory neurons with features characteristic of lateral ganglionic eminence (LGE) origin, 6 clusters of excitatory neurons, glioblasts, astrocytes, oligodendroglial precursors (OPC), ependymal cells, and 1 cluster with mixed identity (Supplemental Table 1, A and B). Of the 3 radial glia clusters, all identified at 5 weeks, *GRN^+/+^* organoids were more enriched in RG1 and RG2, whereas *GRN^–/–^* organoids were more enriched in RG3 (Figure 2, A–C). No differences were noted in several transient cell populations, including the early neuroblasts (cluster 3), CT neuroblasts (cluster 4), or ependymal cells (cluster 16), though a modest increase in OPCs (cluster 15) at 5 weeks in *GRN^+/+^* organoids was noted. Consistent with the data in Figure 1, F and G, *GRN^–/–^* organoids contained more *CTIP2^+^* neurons (cluster 7) than *GRN^+/+^* organoids at 5 weeks (Figure 2, A–C). Compared with *GRN^–/–^* organoids, *GRN^+/+^* organoids had many more *CTIP2^+^SATB2^+^* neurons (clusters 8–9) and *SATB2^+^* neurons (clusters 10–12) at 16 and 25 weeks (Figure 2B). *GRN^–/–^* organoids generated more LGE-type inhibitory neurons (clusters 5–6) at 16 and 25 weeks. Consistent with histological data (Figure 1I), *GRN^–/–^* organoids had significantly more cells in the astrocyte cluster (cluster 14) than *GRN^+/+^* organoids at 16 and 25 weeks (Figure 2, A and B). In addition, *GRN^–/–^* organoids contained more cells with mixed neuron-glia identity (cluster 17) at 16 and 25 weeks. Finally, we projected our datasets onto the cortical organoid datasets previously published by our group (16). As expected, essentially all the major cell clusters identified in our previous cortical organoids, including the radial glia, excitatory neurons, inhibitory neurons, and astrocytes, were represented in both *GRN^+/+^* and *GRN^–/–^* cortical organoids in this study (Figure 2, D and E). Taken together, our scRNA-seq data not only validated the relative abundance of radial glia, excitatory neurons, and astrocytes in *GRN^–/–^* organoids at 16 and 25 weeks but also revealed the expansion of LGE-type inhibitory neurons and the mixed-identity cluster. The presence of LGE-type inhibitory neuron clusters most likely represented the inhibitory neurons that arise de novo in the cerebral cortex (18, 19).

To ensure that our cortical organoids shared the areal identity with the brain regions vulnerable to disease in FTLD, we examined the expression of marker genes that defined different regions in the prenatal human brain (20). This approach revealed that both *GRN^+/+^* and *GRN^–/–^* cortical organoids had high expression of pallium markers *EMX2*, *FOXG1*, and *PAX6* but no expression of subpallium marker *NKX2-1* or midbrain marker *WNT1* (Supplemental Figure 2E). Furthermore, we leveraged regional transcriptomic signatures for prenatal human brain (Supplemental Table 1C) and showed that our cortical organoids exhibited robust transcriptomic profiles resembling the somatosensory cortex but had modest to minimal expression profiles for other cortical regions, including the motor, prefrontal, temporal, parietal, and visual cortex. No gene expression profiles for striatum, thalamus, or cerebellum were identified (Supplemental Figure 2F), though the expression of several FTLD-related genes, including *PSAP*, *TMEM106B*, *GRN*, and *STMN2*, was detected in most cell clusters (Supplemental Figure 2E). These results supported that iPSC-derived cortical organoids are a suitable model to investigate disease mechanisms for FTLD.

### Immune activation and decreased synaptic support in GRN^–/–^ astrocytes.

**Figure 3:**
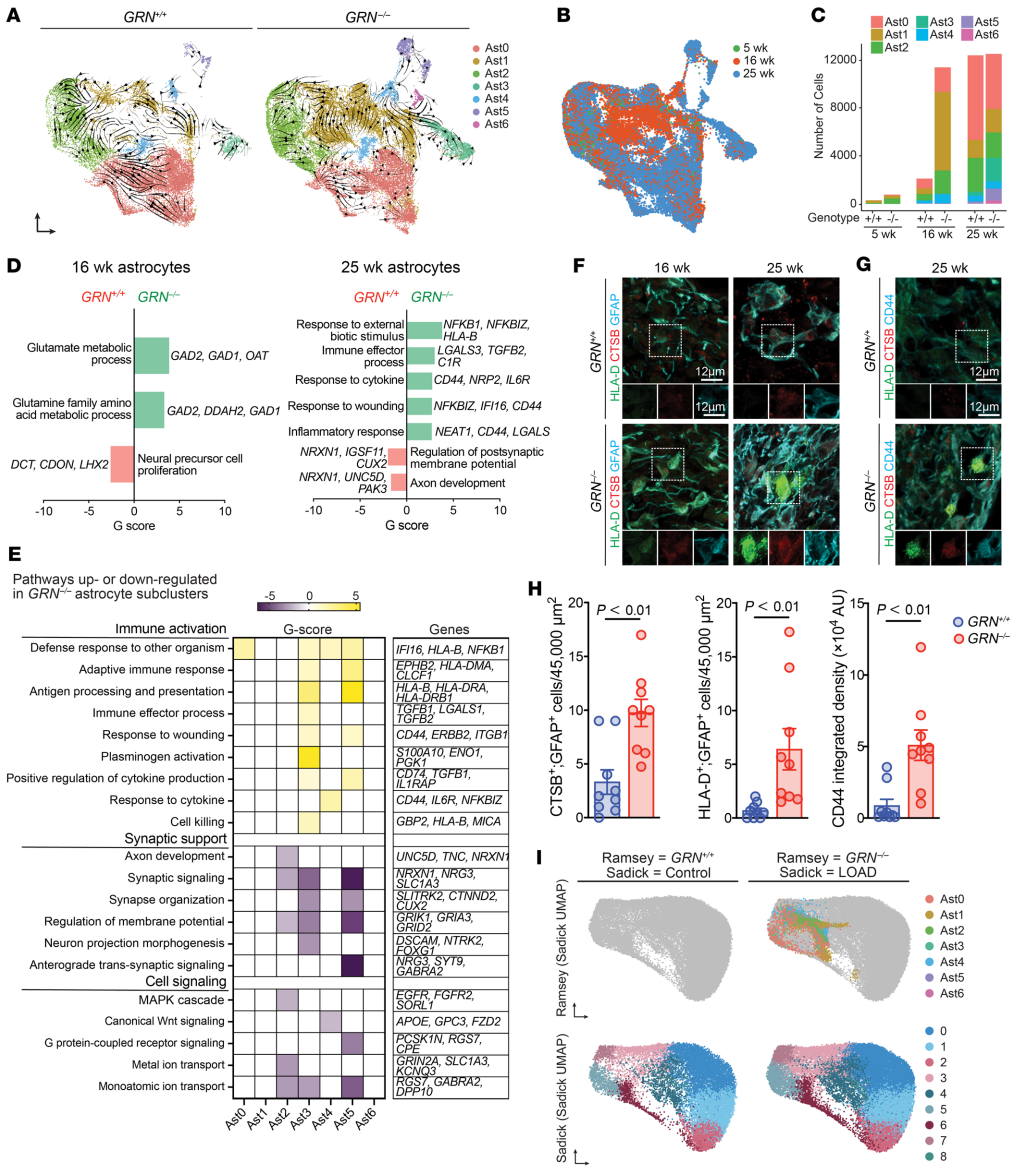
Transcriptomic profiles of astrocyte subclusters in *GRN^+/+^* and *GRN^–/–^* cortical organoids. (**A** and **B**) UMAP of astrocytes grouped by subclusters Ast0-Ast6 split by genotype (**A**) and age (**B**). (**C**) Bar graphs of the total number of cells in each astrocyte subcluster by age and genotype. (**D**) Bar graphs of the top Gene Ontology (GO) terms defined by up- and downregulated genes in *GRN^–/–^* astrocytes compared with *GRN^+/+^* astrocytes in 16- and 25-week organoids. G-score refers to avgFC*-log(adj_*P*_val). (**E**) Heatmap showing the G-scores of top up- and downregulated GO terms in each subcluster of *GRN^–/–^* astroglia compared with *GRN^+/+^* astroglia. Genes listed are top DEGs in the *GRN^–/–^* astroglia in the listed GO term. G-score refers to avgFC*-log(adj_*P*_val). (**F** and **G**) Immunostaining of HLA-D, CTSB, and GFAP (**F**) or HLA-D, CTSB, and CD44 (**G**) in *GRN^+/+^* and *GRN^–/–^* iPSC-derived cortical organoids at 16 and 25 weeks. (**H**) Quantification of CTSB^+^GFAP^+^, HLA-D^+^GFAP^+^, and CD44^+^ astrocytes in *GRN^+/+^* and *GRN^–/–^* cortical organoids at 25 weeks. Statistics used Student’s *t* test; data represent mean ± SEM. (**I**) Top row has Ramsey *GRN^+/+^* and *GRN^–/–^* astroglia clusters projected onto the Sadick UMAPs of control (Ctrl) and Alzheimer’s (AD) astrocytes (25), respectively. Bottom row has Sadick UMAPs of Ctrl and AD astrocytes grouped by Sadick clusters.

Given the robust expansion of *GRN^–/–^* astrocytes in cortical organoids, we asked whether loss of PGRN altered the composition and properties of different astrocyte subtypes. To test this, we combined glioblast and astrocyte clusters and conducted unsupervised reclustering that resulted in 7 subtypes, Ast0–Ast6 (Figure 3A and Supplemental Table 2A). To investigate the identities of these subclusters, we probed the expression of canonical astrocyte markers; reactive astrocyte markers (15); progenitor markers; ER stress–related markers; immune markers, such as the major histocompatibility complex I (MHCI) genes; *CD44*, an activation marker for memory T helper 1 (Th1) cells (21, 22); and FTLD proteinopathy markers (Supplemental Figure 3A and Supplemental Table 2, B and C). This approach revealed that the canonical astrocyte pathways were most highly enriched in Ast0 and Ast4 (Supplemental Figure 3, B and C). Ast2 was enriched in pathways related to cell cycle, indicating that these astrocytes were actively proliferating and thus represented the most immature among all subtypes (Supplemental Figure 3, B and C). Ast1 was enriched in pathways related to translation and mitochondrial function, whereas immune activation pathways were enriched in Ast3, Ast5, and Ast6 (Supplemental Figure 3B). Among the 7 astrocyte subtypes, Ast0, Ast1, and Ast2 were the most enriched at 16 weeks. By 25 weeks, Ast3 and Ast5 showed a significant expansion (Figure 3, B and C). Notably, Ast6 was a small cluster that existed only in *GRN^–/–^*, but not in *GRN^+/+^*, astrocytes (Figure 3, A and C).

To further characterize the stage-dependent transcriptomic changes in *GRN^–/–^* astrocytes, we first combined all astrocyte subclusters and analyzed their differentially expressed genes (DEGs) and pathway enrichment at 16 and 25 weeks. Compared with *GRN^+/+^* astrocytes, *GRN^–/–^* astrocytes were more enriched in pathways related to glutamate metabolism at 16 weeks, whereas by 25 weeks *GRN^–/–^* astrocytes were more enriched in pathways related to immune activation (Figure 3D and Supplemental Table 2, D and E). Next, we analyzed the DEGs and pathway enrichment in each individual astrocyte subcluster and showed that subclusters Ast3 and Ast5 in *GRN^–/–^* astrocytes exhibited significant upregulation in immune activation pathways, such as antigen presentation and adaptive immunity (Figure 3E and Supplemental Table 2, F and G). In support of these results, immunostaining revealed more prominent HLA-D proteins in GFAP^+^ *GRN^–/–^* astrocytes than in *GRN^+/+^* astrocytes at 16 and 25 weeks where no HLA-D was detected (Figure 3, F and H). Like the lysosomal phenotypes in *GRN^–/–^* microglia (7, 23, 24), many *GRN^–/–^* astrocytes contained more abundant lysosomal protein CTSB (Figure 3, F and H), supporting immune activation and potential lysosomal dysfunction in *GRN^–/–^* astrocytes. In addition, *GRN^–/–^* astrocytes also had more abundant CD44 expression than *GRN^+/+^* astrocytes at 25 weeks (Figure 3, G and H). In addition to the upregulated immune response pathways, subclusters Ast2, Ast3, and Ast5 in *GRN^–/–^* astrocytes showed downregulation in pathways related to synaptic support, including synaptic signaling and regulation of membrane potential, whereas subclusters Ast2, Ast3, Ast4, and Ast5 in *GRN^–/–^* astrocytes exhibited downregulation in pathways related to cell signaling (Figure 3E and Supplemental Table 2, F and G). Consistent with these results, confocal microscopy revealed increased neuronal stress and cell death in *GRN^–/–^* organoids at 25 weeks (Supplemental Figure 3, D and E).

Since polymorphisms in *GRN* gene have been implicated as a risk factor for late-onset Alzheimer’s disease (LOAD), we projected the transcriptomes of *GRN^+/+^* and *GRN^–/–^* astrocytes to those from patients with LOAD (25–28) (Figure 3I, Supplemental Figure 4A). This comparison showed that *GRN^–/–^* astrocytes were more enriched with cluster 4 astrocytes of the LOAD dataset, which expressed genes related to cell-matrix adhesion, including CD44 (25). In addition, *GRN^–/–^* astrocytes and LOAD astrocytes shared 187 upregulated genes, most of which were involved in apoptotic process, response to xenobiotic stimulus, autophagy, B cell receptor signaling, innate immune response, and canonical NF-κB signaling (Supplemental Figure 4B and Supplemental Table 2, H and I). Finally, we compared the DEGs in astrocyte clusters in *GRN^–/–^* cortical organoids with DEGs of astrocyte clusters from the frontal cortex of controls and patients with FTLD-GRN and thalami of 12- and 19-month-old *Grn^+/+^* and *Grn^–/–^* mice (15, 24). These comparisons revealed significant overlaps in DEGs in astrocyte clusters across *GRN^–/–^* cortical organoids, frontal cortex from patients with FTLD-GRN, and thalami from *Grn^–/–^* mice (Supplemental Figure 4, C and D). Collectively, these results support the highly conserved astroglial phenotypes caused by PGRN deficiency across different species.

### Activation of TGF-β signaling pathway in GRN^–/–^ astrocytes.

**Figure 4:**
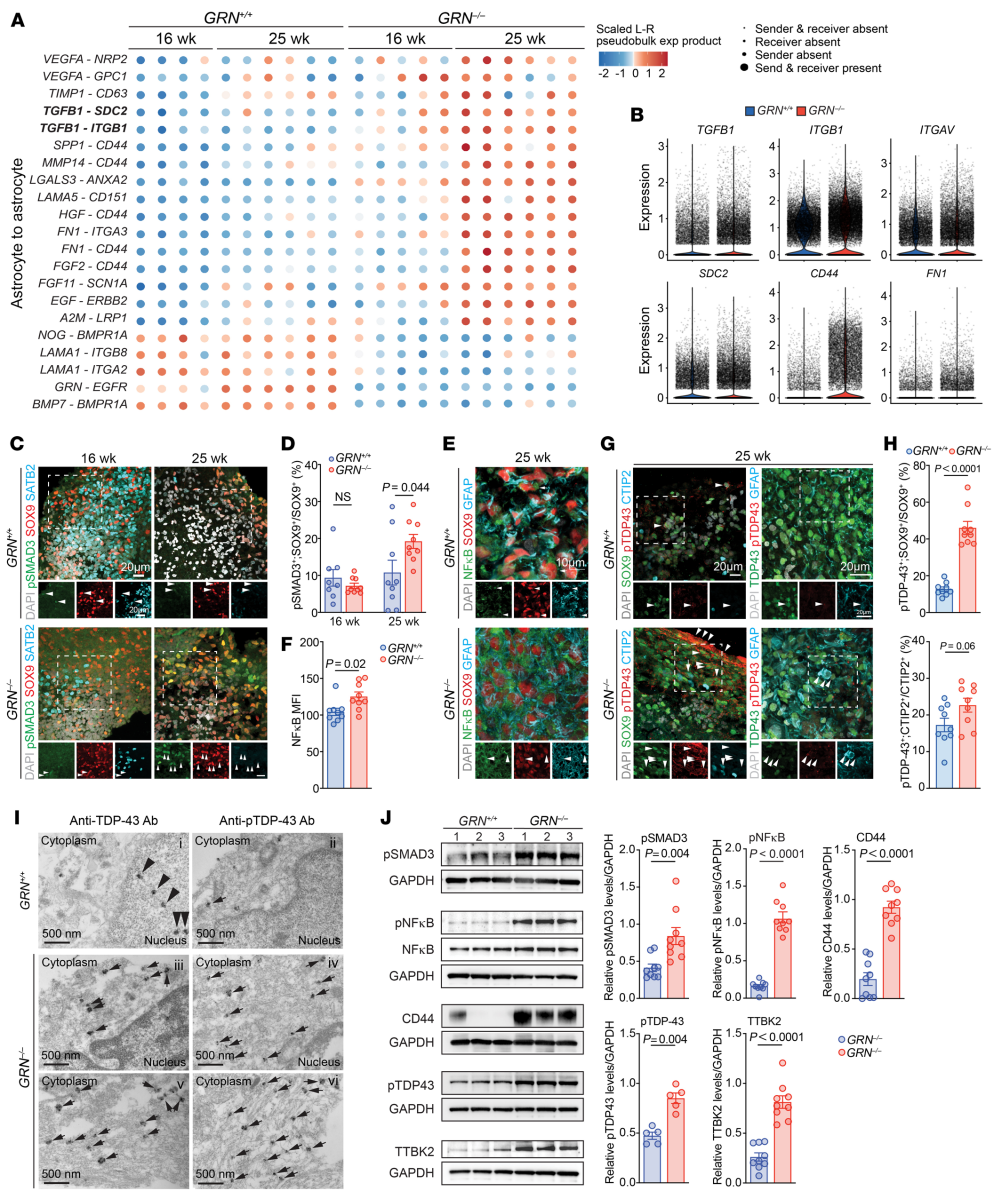
MultiNicheNet analyses of the signaling mechanisms mediating cell-cell communications in *GRN^+/+^* and *GRN^–/–^* cortical organoids. (**A**) Heatmap of astrocyte-to-astrocyte ligand-receptor pairs split by age and genotype. (**B**) Violin plots for the expression of *TGFB1*, *ITGB1*, *ITGAV*, *SDC2*, *CD44*, and *FN1* in *GRN^+/+^* and *GRN^–/–^* astrocytes. Data represent mean + SEM. (**C** and **D**) Confocal images and quantification of SOX9^+^pSMAD3^+^ cells in *GRN^+/+^* and *GRN^–/–^* cortical organoids at 25 weeks. (**E** and **F**) Confocal images and quantification of NF-κB mean fluorescence intensity (MFI) in *GRN^+/+^* and *GRN^–/–^* iPSC-derived cortical organoids at 25 weeks. (**G** and **H**) Confocal images and quantification of pTDP-43^+^SOX9^+^ cells in *GRN^+/+^* and *GRN^–/–^* cortical organoids at 25 weeks. In addition, confocal images of TDP-43, pTDP-43, and GFAP in 25 weeks *GRN^+/+^* and *GRN^–/–^* organoids show nuclear depletion of TDP-43 and the presence cytoplasmic pTDP-43 in GFAP^+^ *GRN^–/–^* astrocytes. All data represent mean ± SEM. (**I**) Immuno-gold electron microscopy (IEM) of total TDP-43 (left column) and pTDP-43 (right column) in *GRN^+/+^* and *GRN^–/–^* cortical organoids at 25 weeks. Arrows in first image highlight TDP-43^+^ structures in the nucleus of a *GRN^+/+^* astrocyte, whereas arrowheads highlight many total TDP-43^+^ and pTDP-43^+^ structures in *GRN^–/–^* astrocytes (middle). (**J**) Western blots and quantification of the relative abundance of pSMAD3, pNF-κB, CD44, pTDP-43, and TTBK2 in *GRN^+/+^* and *GRN^–/–^* cortical organoids at 25 weeks. Statistics used Student’s *t* test; data represent mean ± SEM.

To investigate how *GRN^–/–^* astrocytes impacted other cell types in the cortical organoids, we performed MultiNicheNet analyses (29), focusing on the top 50 signaling pathways mediating cell-cell communications via ligand-receptor pairing and downstream signaling targets among astrocytes, radial glia, *CTIP2^+^* neurons, *CTIP2^+^SATB2^+^* neurons, *SATB2^+^* neurons, and inhibitory neurons at 16 and 25 weeks. Since *CTIP2^+^* neurons and *CTIP2^+^SATB2^+^* neurons did not show any significant differences in interaction, they were excluded from the final analyses. In *GRN^+/+^* organoids, astrocytes and *SATB2^+^* neurons used a range of signaling pathways to communicate within themselves and with each other, including using *GRN*-*EGFR* as a cell-autonomous signal in astrocytes. Only very limited ligand-receptor pairs were identified between astrocytes and *SATB2^+^* neurons and inhibitory neurons. In *GRN^–/–^* organoids, however, astrocytes underwent a drastic expansion of signaling pathways to astrocytes themselves and to inhibitory neurons (Supplemental Figure 5A). Among the signaling pathways, *TGFB1* and *EGF* signals appeared to act cell-autonomously on *GRN^–/–^* astrocytes by interacting with *SDC2* and *ITGB1*, and with *ERBB2*, respectively. In support of these results, gene network interaction revealed several prominent “interacting nodes” in *GRN^–/–^* astrocytes with *TGFB1* as the most prominent node (Supplemental Figure 5B). Since ITGB1 has been known to dimerize with ITGAV to activate TGF-β (30, 31), we examined the expression of ITGAV using immunostains for ITGAV, SOX9, and SATB2 in organoids at 16 and 25 weeks. These results showed elevated ITGAV expression in *GRN^–/–^* organoids at both 16 and 25 weeks, especially in SOX9^+^ cells (Supplemental Figure 3F). Unlike *GRN^+/+^* astrocytes, *GRN^–/–^* astrocytes showed downregulation in *LAMA1*-*ITGA2*/*ITGB8* and *BMP7*-*BMPRA1* pathways and progressive upregulation in *VEGFA-GPC1*, *TGFB1-ITGB1*, *SPP1-CD44*, and *FN1-CD44* signaling from 16 weeks to 25 weeks (Figure 4, A and B). In addition, *GRN^–/–^* astrocytes also deployed multiple ligands that converged on *GRN^–/–^* radial glia via *ITGB1*, *EGFR*, and *APLP2* (amyloid beta precursor like protein 2) (Supplemental Figure 3G).

To further characterize the aberrant activation of TGF-β1 signaling in *GRN^–/–^* astrocytes and how this might impact their phenotypes, we used pSMAD3 and NF-κB as markers for the canonical and noncanonical TGF-β signaling pathways, respectively (32). Using confocal microscopy, we showed that *GRN^+/+^* organoids contained very few pSMAD3^+^SOX9^+^ cells at 16 and 25 weeks (Figure 4, C and D). Like *GRN^+/+^* organoids, *GRN^–/–^* organoids contained few pSMAD3^+^SOX9^+^ cells at 16 weeks. By 25 weeks, however, significantly more pSMAD3^+^SOX9^+^ cells were present in *GRN^–/–^* organoids, many with intense pSMAD3 nuclear staining (Figure 4, C and D). No pSMAD3^+^ was detected in SATB2^+^ neurons in *GRN^+/+^* or *GRN^–/–^* organoids at both stages (Figure 4C). Similar to pSMAD3, many SOX9^+^ cells in *GRN^–/–^* organoids showed robust NF-κB and GFAP staining at 25 weeks (Figure 4, E and F). Finally, we asked whether *GRN^–/–^* organoids could recapitulate TDP-43 proteinopathy, a diagnostic feature of FTLD-GRN (2, 13). While *GRN^+/+^* organoids had rare pTDP-43^+^ cells at 16 and 25 weeks, *GRN^–/–^* organoids had significantly more pTDP-43^+^ cells at both ages (Figure 4, G and H). To determine which cells contained these pTDP-43^+^ fibrils, we stained *GRN^+/+^* and *GRN^–/–^* organoids with SOX9, pTDP-43, and CTIP2 antibodies or with TDP-43, pTDP-43, and GFAP antibodies. In *GRN^+/+^* organoids, SOX9^+^ or GFAP^+^ cells contained no detectable pTDP-43 immunofluorescent signals in the cytoplasm (Figure 4G). In contrast, *GRN^–/–^* organoids contained a thick layer of pTDP-43^+^ granules or fibrils, especially at the outer edge of the organoids (Figure 4G). Some pTDP-43 in *GRN^–/–^* organoids was identified in the cytoplasm of GFAP^+^ astrocytes that had lost their nuclear TDP-43 (Figure 4G). To investigate the ultrastructural characteristics of these pTDP-43^+^ structures, we performed immuno-gold electron microscopy (IEM) in 25-week *GRN^+/+^* and *GRN^–/–^* cortical organoids using antibodies for total TDP-43 or pTDP-43. These results showed that in *GRN^+/+^* organoids TDP-43 proteins were detected in the nuclei of astrocytes, whereas only very few pTDP-43 proteins were detected in the cytoplasm (Figure 4I, top). In contrast, astrocytes in *GRN^–/–^* cortical organoids contained TDP-43^+^ and pTDP-43^+^ proteins, and many were embedded within granules or fibril-like structures (Figure 4I, middle). Together, these IEM results confirmed the results from confocal microscopy and supported that PGRN deficiency in cortical organoids can lead to mislocalization and aggregation of pTDP-43 protein in astrocytic cytoplasm. To validate the phenotypes in *GRN^–/–^* cortical organoids, we performed Western blots and confirmed the significant upregulation of pSMAD3, pNF-κB, CD44, and pTDP-43 in *GRN^–/–^* cortical organoids (Figure 4J). In addition, several kinases have been implicated in the phosphorylation of cytoplasmic TDP-43, including casein kinase 1/2 (33), CDC7 (34), tau-tubulin kinase 1/2 (TTBK1/2) (35), and IκB kinase (36). To this end, we leveraged our scRNA-seq datasets to show that *TTBK2* and *CDC7* were more abundant in *GRN^–/–^* astrocytes than *GRN^+/+^* astrocytes, whereas *TTBK1* showed much lower expression (Supplemental Figure 6, A–F). Consistent with these results, Western blots showed that the protein level of TTBK2 was upregulated in *GRN^–/–^* cortical organoids (Figure 4L). In contrast, CDC7 protein levels showed no difference, whereas TTBK1 proteins were below detectable levels, between *GRN^+/+^* and *GRN^–/–^* cortical organoids (Supplemental Figure 6, G and H).

**Figure 5:**
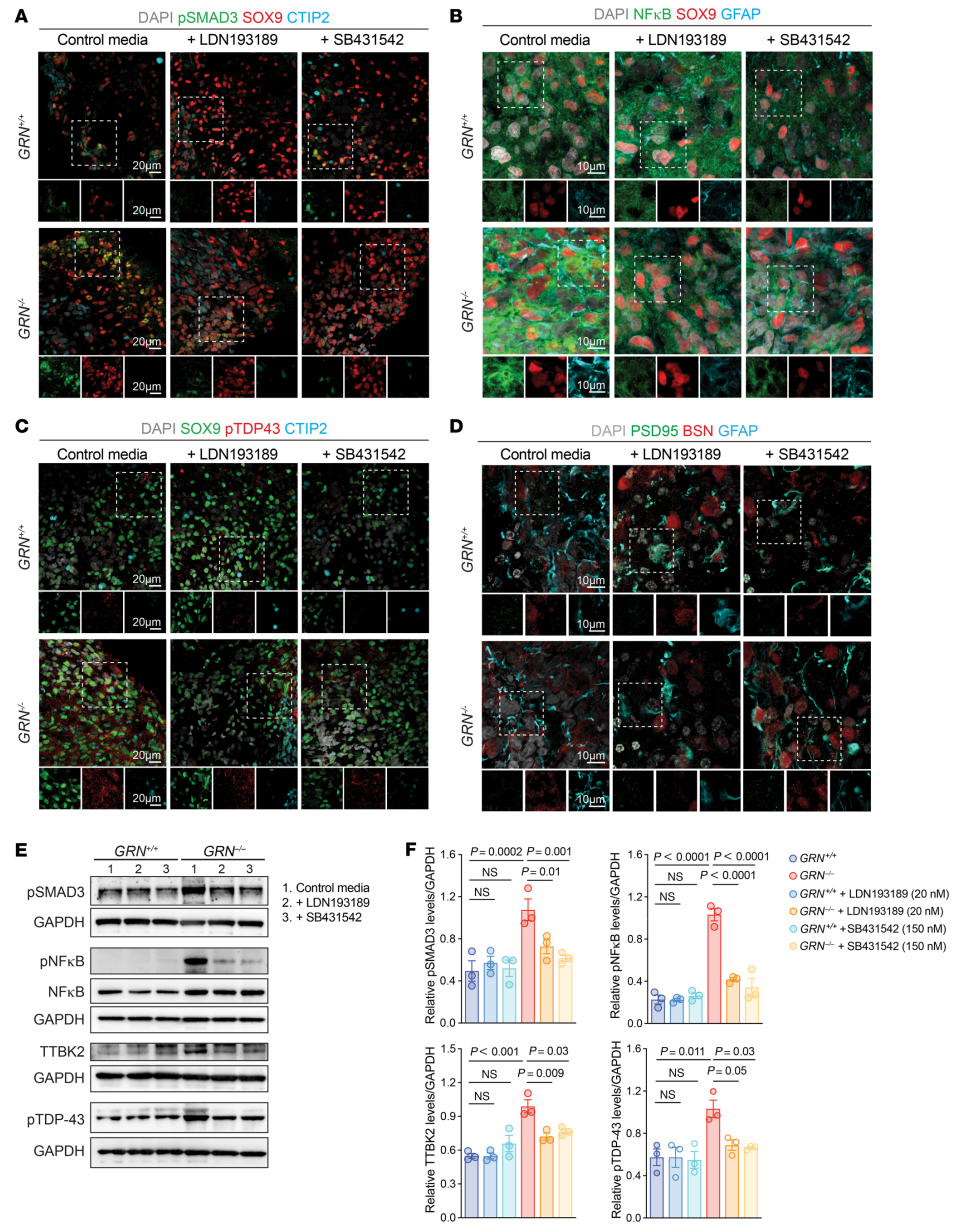
Inhibition of TGF-β signaling reduces pTDP-43 in *GRN^–/–^* astrocytes and restores synaptic density in *GRN^–/–^* cortical organoids. (**A**–**D**) Immunostaining of pSMAD3, SOX9, CTIP2 (**A**); NF-κB, SOX9, GFAP (**B**); SOX9, pTDP-43, CTIP2 (**C**); and PSD95, Bassoon (BSN), GFAP (**D**) in *GRN^+/+^* and *GRN^–/–^* cortical organoids cultured under control media or treated with TGF-β receptor inhibitor LDN193189 (20 nM) or SB431542 (150 nM) from 20 to 25 weeks and harvested for histopathological characterizations at 25 weeks. (**E** and **F**) Western blots and quantification of the relative abundance of pSMAD3, pNF-κB, TTBK2, and pTDP-43 in *GRN^+/+^* and *GRN^–/–^* cortical organoids cultured under control media or treated with LDN193189 or SB431542 from 20 to 25 weeks. Organoids (*n* = 3) from 3 independent biological replicates. All data represent mean ± SEM. Statistics used 2-way ANOVA with multiple comparisons.

Given the robust upregulation of pSMAD3 and NF-κB in *GRN^–/–^* astrocytes, we asked whether activation of TGF-β signaling might be responsible for the increased pTDP-43 in *GRN^–/–^* organoids. To test this, we treated *GRN^+/+^* and *GRN^–/–^* organoids with TGF-β receptor inhibitors, LDN193189 (20 nM) or SB431542 (150 nM), from 20 to 25 weeks. In *GRN^–/–^* organoids, both inhibitors significantly reduced the intensity of NF-κB^+^ staining, the percentage of pSMAD3^+^SOX9^+^ cells, pTDP-43^+^ fibrils, and HLA-D expression in *GRN^–/–^* astrocytes (Figure 5, A–D, and Supplemental Figure 7A). Both TGF-β receptor inhibitors also restored the synaptic density in *GRN^–/–^* organoids and reduced cellular stress (Figure 5, D and E, and Supplemental Figure 7, B–D). Western blot analyses confirmed the effects of TGF-β receptor inhibitors to rescue pSMAD3, pNF-κB, and upregulation of TTBK2 in *GRN^–/–^* organoids (Figure 5, E and F). In contrast, the same treatment in *GRN^–/–^* organoids did not reduce the number of pTDP-43^+^CTIP2^+^ cells, nor did it affect the number of SOX9^+^ cells or apoptotic cell death in *GRN^–/–^* organoids (Supplemental Figure 7, A–D). These results support the importance of aberrant TGF-β signaling in promoting immune activation and TDP-43 proteinopathy in *GRN^–/–^* astrocytes. Finally, we treated *GRN^+/+^* and *GRN^–/–^* organoids with recombinant PGRN from 16 to 25 weeks and showed that PGRN replacement significantly reduced the number of pSMAD3^+^SOX9^+^, NF-κB^+^SOX9^+^, and pTDP-43^+^SOX9^+^ cells in *GRN^–/–^* organoids (Supplemental Figure 8). Together, these results support that activation of TGF-β pathway is key to the phenotypes observed in astrocytes in *GRN^–/–^* organoids.

**Figure 6:**
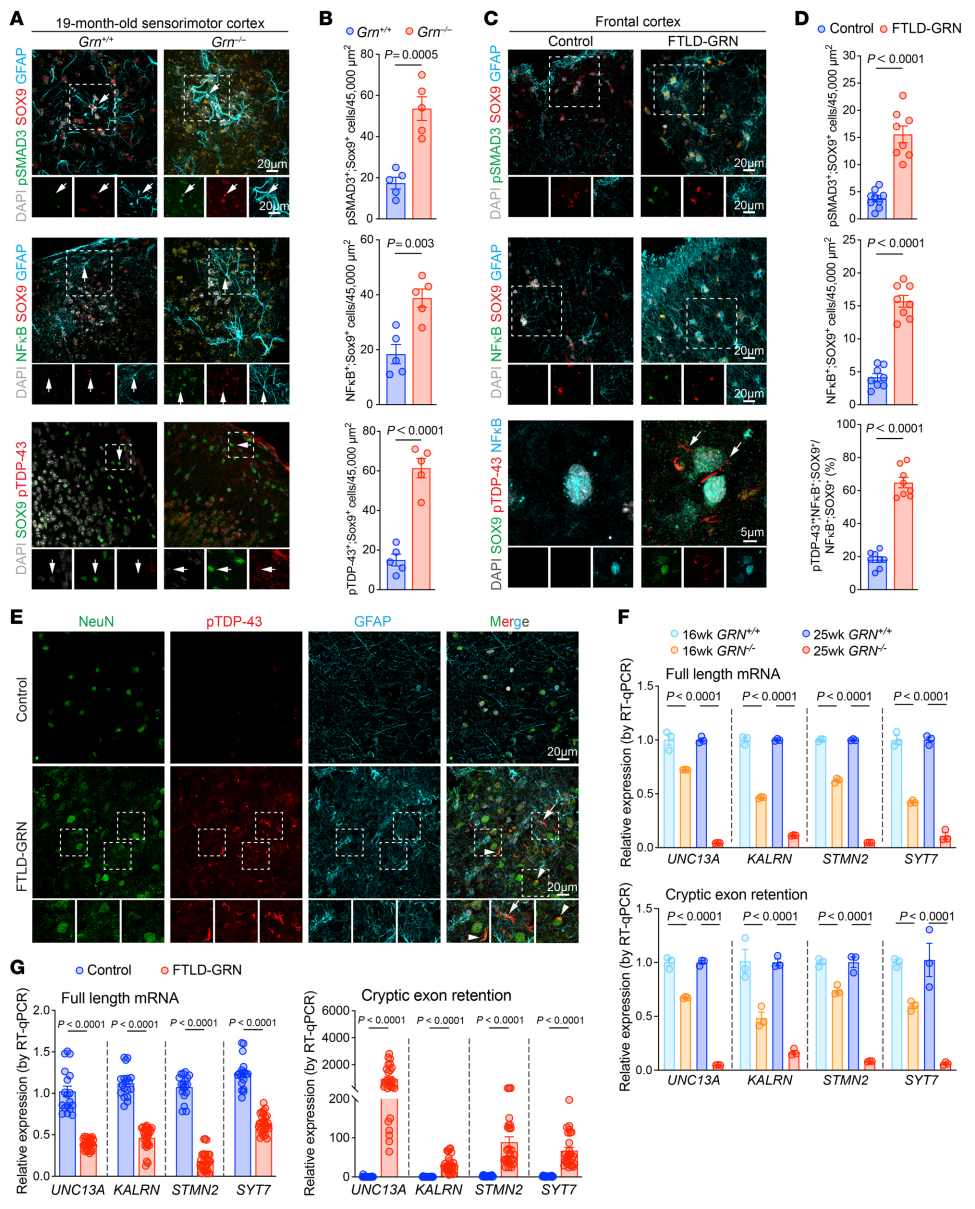
Conserved TGF-β activation in PGRN-deficient astrocytes is independent of cryptic exon retention phenotype. (**A**) Confocal images of pSMAD3, SOX9, and GFAP (upper row); NF-κB, SOX9, and GFAP (middle row); and pTDP-43 and Sox9 (lower row) in the sensorimotor cortex of 19-month-old *Grn^+/+^* and *Grn^–/–^* mice. (**B**) Quantification of pSMAD3^+^Sox9^+^, NF-κB^+^Sox9^+^, and pTDP-43^+^Sox9^+^ astrocytes in sensorimotor cortex of 19-month-old *Grn^+/+^* and *Grn^–/–^* mice. (**C**) Confocal images of pSMAD3, SOX9, and GFAP (upper row); NF-κB, SOX9, and GFAP (middle row); and pTDP-43 and SOX9 (lower row) in the frontal cortex of controls and patients with FTLD-GRN. (**D**) Quantification of pSMAD3^+^SOX9^+^, NF-κB^+^SOX9^+^, and pTDP-43^+^SOX9^+^ astrocytes in frontal cortex of controls and patients with FTLD-GRN. *N* = 8 for each group; data represent mean ± SEM. Statistics used Student’s *t* test. (**E**) Confocal images of NeuN, pTDP-43, GFAP, and DAPI in the middle frontal gyrus of control and FTLD-GRN patients. Insets in the bottom panels from FTLD-GRN frontal cortex highlight NeuN+;pTDP-43+ fibril (left panel, pTDP-43+;GFAP+ fibrils (middle panel), and cytoplasmic pTDP-43+ aggregate in NeuN+ neuron (right panel). (**F** and **G**) Quantification of the relative abundance (upper graphs) and cryptic exon retention (lower graphs) of UNC13A, KALRN, STMN2, and SYT7 in GRN+/+and GRN-/- cortical organoids at 16 and 25 weeks (**E**) and in the frontal cortex of control and FTLD-GRN patients (**F**). Data represent mean ± SEM. Statistics used two-way ANOVA with multiple comparisons.

Given the significant overlap in the transcriptomic profiles in astrocytes in *GRN^–/–^* cortical organoids, *Grn^–/–^* mice, and patients with FTLD-GRN (Supplemental Figure 4, C and D), we asked whether similar histopathological features in astrocytes of *GRN^–/–^* organoids could be identified in *Grn^–/–^* mice and patients with FTLD-GRN. Using confocal microscopy, we showed that astrocytes in the sensorimotor cortex of 19-month-old *Grn^–/–^* mice and frontal cortex of patients with FTLD-GRN also exhibited robust expression of pSMAD3, NF-κB, and pTDP-43 (Figure 6, A–D). Next, we asked whether *GRN^–/–^* cortical organoids also exhibited cryptic exon retention as seen in patients with FTLD-TDP (37, 38). Using reverse transcription quantitative PCR (RT-qPCR), we showed that the genes implicated in cryptic exon retention, such as *UNC13A*, *KALRN*, *STMN2*, *SYT7*, were significantly downregulated in *GRN^–/–^* cortical organoids at 16 and 25 weeks and in the frontal cortex of patients with FTLD-GRN (Figure 6, E and F). Consistent with these results, scRNA-seq data from *GRN^+/+^* and *GRN^–/–^* organoids revealed that *KALRN* and *STMN2* transcripts were modestly downregulated in several neuronal clusters, e.g., LGE inhibitory neurons and CTIP2 and SATB2 neurons, whereas no significant downregulation of *SYT7* or *UNC13A* was observed (Supplemental Figure 9A). However, while the frontal cortex of patients with FTLD-GRN showed robust cryptic exon retention in *UNC13A*, *KALRN*, *STMN2*, and *SYT7*, no cryptic exon retention in these genes was detected in *GRN^–/–^* cortical organoids after 16 or 25 weeks in cultures (Figure 6G). The lack of cryptic exon retention phenotypes in *GRN^–/–^* cortical organoids indicated that the culture conditions for cortical organoids may need to be optimized to recapitulate loss of TDP-43 in neurons, which promotes splicing defects seen in the aging brain (38) and in iPSC-derived motor neurons (37).

### GRN^R493X^ cortical organoids phenocopy astroglial phenotypes in GRN^–/–^ cortical organoids.

**Figure 7:**
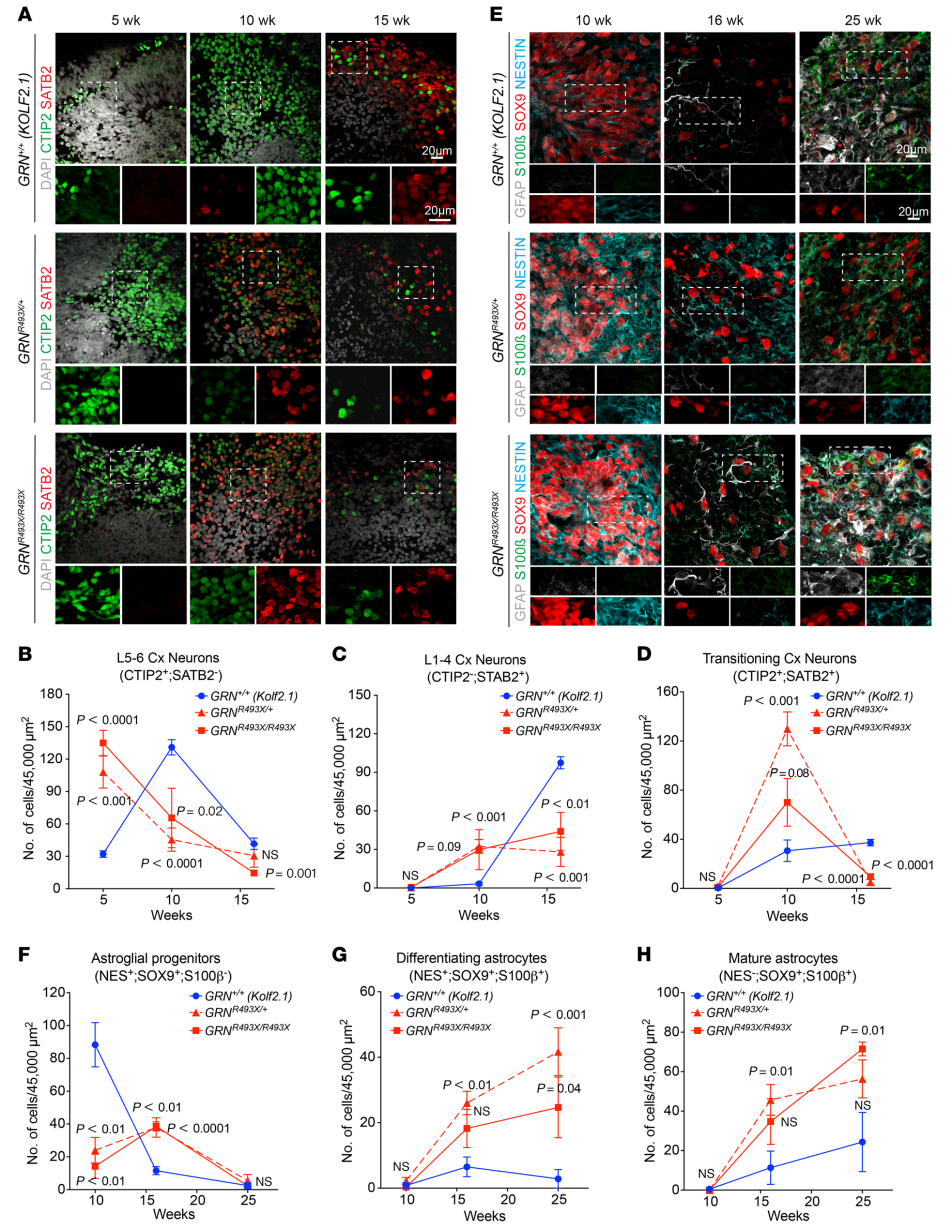
Precocious neurogenesis and astrogliogenesis in *GRN^R493X/R493X^* cortical organoids. (**A**) Confocal images of CTIP2, SATB2, and DAPI in *GRN^+/+^*, *GRN^R493X/+^*, and *GRN^R493X/R493X^* cortical organoids at 5, 10, and 15 weeks. (**B**–**D**) Quantification of the number of CTIP2^+^SATB2^–^ L5–6 cortical neurons (**B**), CTIP2^–^SATB2^+^ L1–4 cortical neurons (**C**), and CTIP2^+^SATB2^+^ transitioning cortical neurons (**D**) at different time points in *GRN^+/+^* (blue circle), *GRN^R493X/+^* (red triangle), and *GRN^R493X/R493X^* (red square) cortical organoids at 5, 10, and 15 weeks. Organoids (*n* = 9) from 3 independent biological replicate experiments were analyzed per time point per genotype. (**E**) Confocal images of NESTIN, SOX9, GFAP, and S100b in *GRN^+/+^*, *GRN^R493X/+^* and *GRN^R493X/R493X^* cortical organoids at 10, 16, and 25 weeks. (**F**–**H**) Quantification of the number of NES^+^SOX9^+^S100b^–^ astroglial progenitors (**F**), NES^+^SOX9^+^S100b^+^ differentiating astrocytes (**G**), and NES^–^SOX9^+^S100b^+^ mature astrocytes (**H**) at different time points in *GRN^+/+^* (blue circle), *GRN^R493X/+^* (red triangle), and *GRN^R493X/R493X^* (red square) cortical organoids at 10, 16, and 25 weeks. Organoids (*n* = 9) from 3 independent biological replicate experiments were analyzed per time point per genotype. Statistics used Student’s *t* test. All data represent mean ± SEM.

To determine whether the astroglial phenotypes in *GRN^–/–^* cortical organoids also exist in cortical organoids that contain human disease–relevant mutations, we obtained CRISPR-engineered iPSCs that carry humanized *GRN^R493X^* mutation (39), including *GRN^R493X/+^* and *GRN^R493X/R493X^* iPSCs and their isogenic control *GRN^+/+^* (*Kolf2.1J*). We used the same conditions in Figure 1A to prepare *GRN^+/+^* (*Kolf2.1J*), *GRN^R493X/+^*, and *GRN^R493X/R493X^* cortical organoids and collected them for morphological analyses at 5, 10, 16, and 25 weeks. Using antibodies for CTIP2 and SATB2, we showed that, compared with *GRN^+/+^* (*Kolf2.1J*) cortical organoids, *GRN^R493X/+^* and *GRN^R493X/R493X^* cortical organoids exhibited significantly more CTIP2^+^SATB2^–^ L5–6 cortical neurons at 5 weeks (Figure 7, A and B). In addition, *GRN^R493X/+^* and *GRN^R493X/R493X^* cortical organoids contained more CTIP2^–^SATB2^+^ L1–4 cortical neurons and CTIP2^+^SATB2^+^ transitioning cortical neurons at 10 weeks (Figure 7, C and D). Using markers for astrocytes, including SOX9, S100b, and GFAP, we further showed that *GRN^R493X/+^* and *GRN^R493X/R493X^* cortical organoids consistently had more astroglial progenitors at 16 weeks and more differentiating astrocytes and mature astrocytes at 16 and 25 weeks (Figure 7, E–H). Together, these results support that, like *GRN^–/–^* cortical organoids (Figure 1), both *GRN^R493X/+^* and *GRN^R493X/R493X^* cortical organoids exhibited similar precocious neurogenesis and astrogliogenesis.

**Figure 8:**
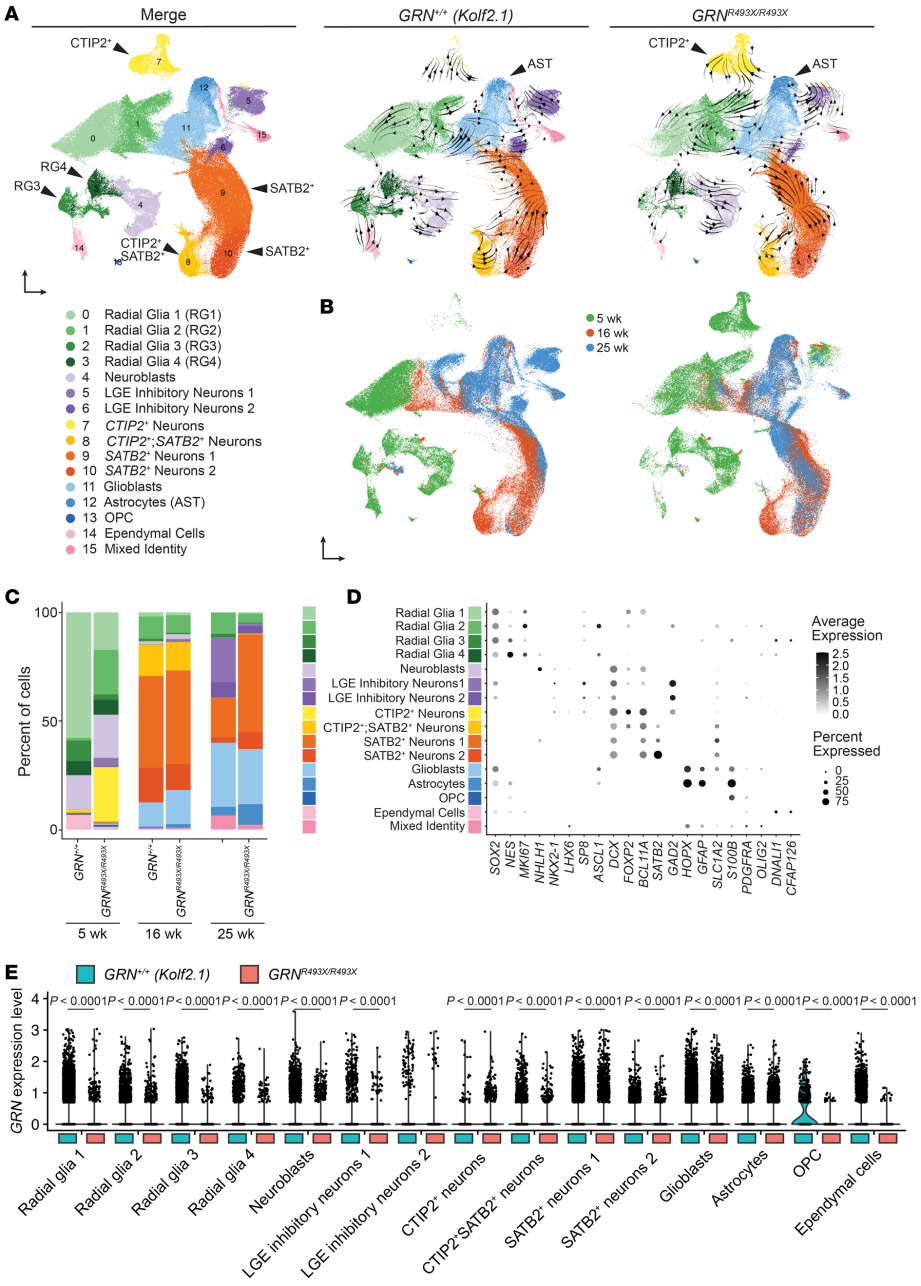
Single-cell transcriptomics of *GRN^+/+^* and *GRN^R493X/R493X^* cortical organoids. (**A**) UMAPs of combined (left), *GRN^+/+^* (center), and *GRN^R493X/R493X^* (right) cortical organoids grouped by cluster with an overlay of RNA velocity vectors. (**B**) UMAPs of *GRN^+/+^* (left) and *GRN^R493X/R493X^* (right) cortical organoids grouped by age. (**C**) Bar graphs of the percentage of cells in each cluster split by age and genotype. (**D**) Dot plots of representative genes used to identify each cluster. (**E**) Violin plots show the expression of GRN transcripts in all cell clusters in control and GRNR493X/R493X organoids.

Given the remarkably similar phenotypes in *GRN^R493X/+^* and *GRN^R493X/R493X^* cortical organoids, we performed scRNA-seq using *GRN^+/+^* (*Kolf2.1J*) and *GRN^R493X/R493X^* cortical organoids at 5, 16, and 25 weeks (Figure 8, A and B). In total, we used 4 biological replicates and 2 experimental replicates for each time point and genotype. After quality control, we obtained 30,343–46,141 cells per time point/genotype, with 4,076 reads and 1,982 genes per cell (Supplemental Figure 10). Unsupervised clustering resulted in 18 clusters, including 4 clusters of radial glia, neuroblasts, 2 clusters of LGE inhibitory neurons, 4 clusters of excitatory neurons, glioblasts, astrocytes, OPCs, ependymal cells, and 1 cluster with mixed identity (Supplemental Table 3, A and B). Similar to *GRN^+/+^* and *GRN^–/–^* cortical organoids, all 4 radial glia clusters were identified at 5 weeks (Figure 8, A and B). Consistent with the data in Figure 7, A and B, *GRN^R493X/R493X^* organoids contained more *CTIP2^+^* neurons (cluster 7) than *GRN^+/+^* organoids at 5 weeks (Figure 8, A–C). Compared with *GRN^+/+^* organoids, *GRN^R493X/R493X^* organoids had more *SATB2^+^* neurons (clusters 9–10) at 25 weeks (Figure 8, A–C). Consistent with histological data (Figure 7, E–H), *GRN^R493X/R493X^* organoids had significantly more cells in the astrocyte cluster (cluster 12) than *GRN^+/+^* organoids at 16 and 25 weeks (Figure 8, C –E).

**Figure 9:**
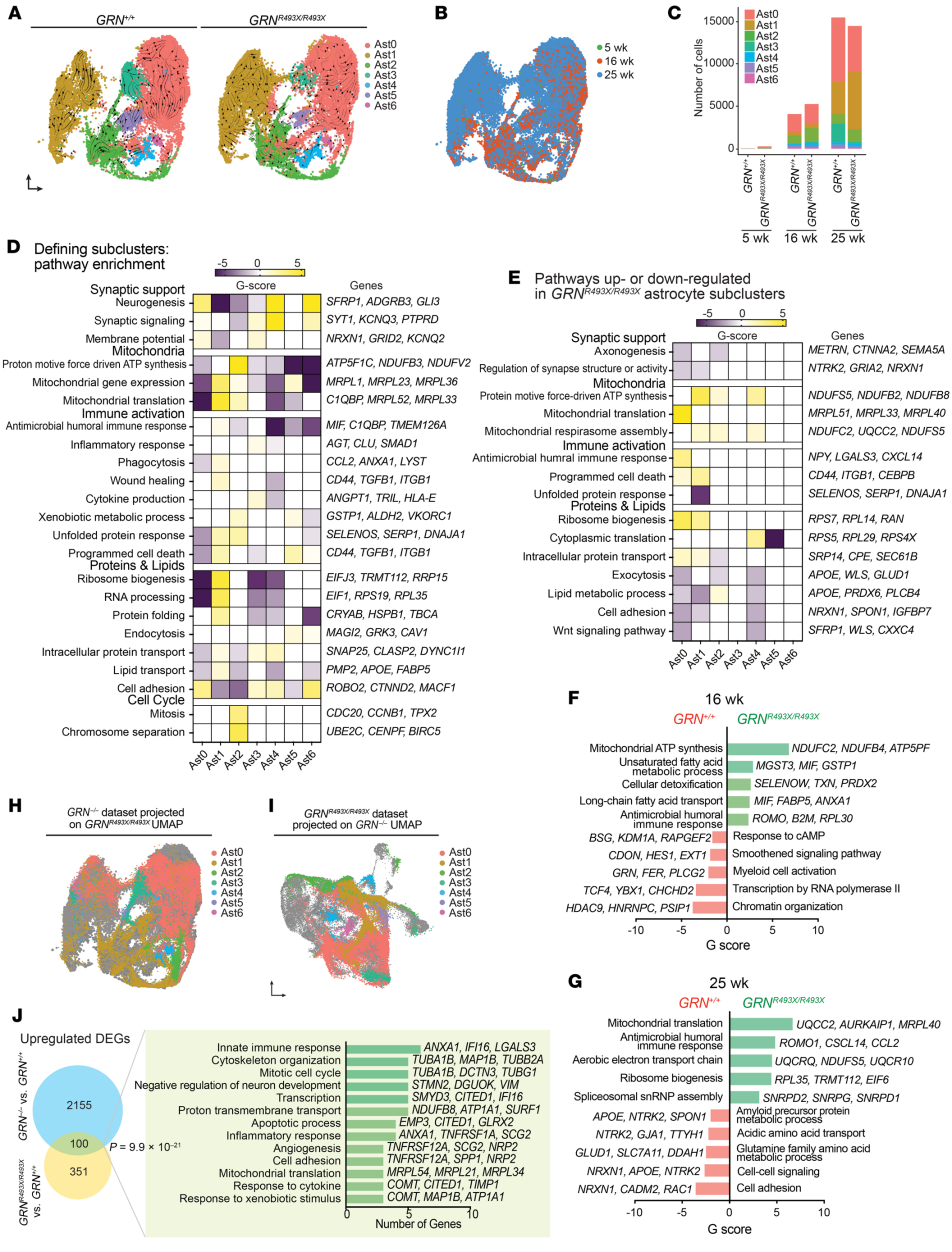
Transcriptomic profiles of astrocyte subclusters in *GRN^+/+^* and *GRN^R493X/R493X^* cortical organoids. (**A** and **B**) UMAP of astrocyte subclusters 0 to 6 (Ast0_R-Ast6_R) split by genotype (**A**) and by age (**B**). (**C**) Bar graphs of the total number of cells in each astrocyte subcluster by age and genotype. (**D**) Heatmap showing the G-scores of top up- and downregulated GO terms in each subcluster compared with the other subclusters. Genes listed are top DEGs in the listed GO term. (**E**) Heatmap showing the G-scores of top up- and downregulated GO terms in each subcluster of *GRN^R493X/R493X^* astrocytes compared with *GRN^+/+^* astrocytes. Genes listed are among the top DEGs in *GRN^R493X/R493X^* astrocytes in the listed GO term. G-score in **D** and **E** refers to avgFC*-log(adj_*P*_val). (**F** and **G**) Bar graphs of the top GO terms defined by up- and downregulated genes in *GRN^R493X/R493X^* astrocytes compared with *GRN^+/+^* astroglia in 16- and 25-week organoids. Genes listed are top DEGs in *GRN^R493X/R493X^* astrocytes. G-score refers to avgFC*-log(adj_*P*_val). (**H**) *GRN^+/+^* and *GRN^–/–^* astrocyte clusters projected onto the *GRN^+/+^* and *GRN^R493X/R493X^* astroglia UMAP. (**I**) *GRN^+/+^* and *GRN^R493X/R493X^* astrocyte clusters projected onto the *GRN^+/+^* and *GRN^–/–^* astrocyte UMAP. (**J**) Venn diagram showing the overlap of the upregulated DEGs between *GRN^–/–^* vs. *GRN^+/+^* and *GRN^R493X/R493X^* vs. *GRN^+/+^* datasets with hypergeometric *P* value. Right panel is a bar graph showing GO terms based on the overlapping DEG list (left).

**Figure 10:**
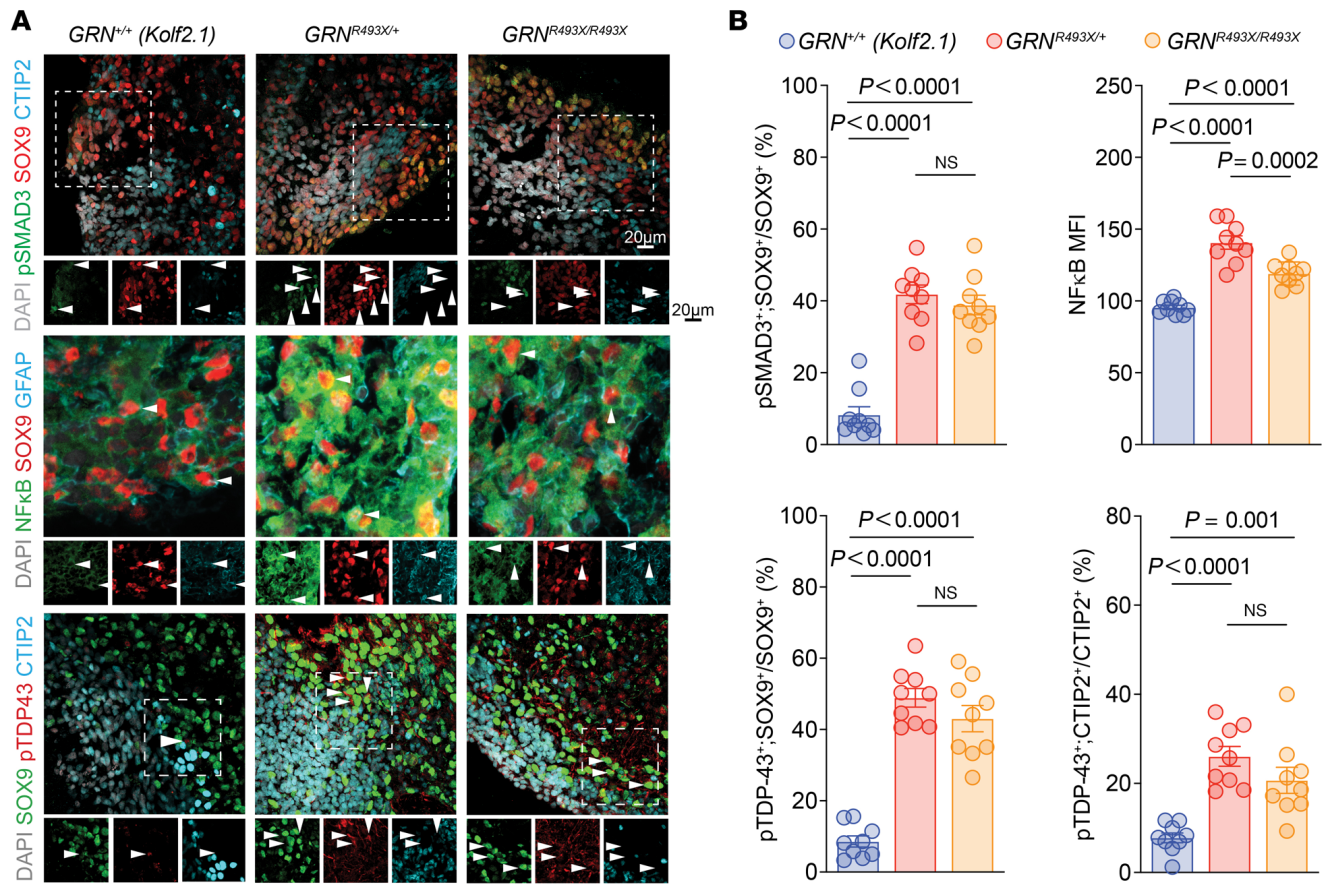
TGF-β activation and pTDP-43 in GRN-mutant astrocytes in *GRN^R493X/+^* and *GRN^R493X/R493X^* cortical organoids. (**A**) Confocal microscopic images of pSMAD3, SOX9, and CTIP2 (upper row); NF-κB, SOX9, and GFAP (middle row); and SOX9, pTDP-43, and CTIP2 (lower row) in *GRN^+/+^*, *GRN^R493X/+^*, and *GRN^R493X/R493X^* cortical organoids at 25 weeks. (**B**) Quantification of pSMAD3^+^SOX9^+^ cells, NF-κB MFI, pTDP-43^+^SOX9^+^ cells, and pTDP-43^+^CTIP2^+^ cells in *GRN^+/+^*, *GRN^R493X/+^*, and *GRN^R493X/R493X^* cortical organoids. All data represent mean ± SEM. Statistics used 2-way ANOVA with multiple comparisons.

Next, we combined glioblast and astrocyte clusters in *GRN^+/+^* (*Kolf2.1J*) and *GRN^R493X/R493X^* cortical organoids and used unsupervised reclustering to identify 7 subtypes, Ast0–Ast6, which emerged primarily at 16 and 25 weeks (Figure 9, A–C, and Supplemental Table 4A). Similar to *GRN^+/+^* and *GRN^–/–^* cortical organoids, *GRN^+/+^* (*Kolf2.1J*) and *GRN^R493X/R493X^* cortical organoids expressed canonical astrocyte markers; reactive astrocyte markers (15); progenitor markers; ER stress–related markers; immune markers, such as the MHCI genes; *CD44*, an activation marker for memory Th1 cells (21, 22); and FTLD proteinopathy markers (Supplemental Figure 11 and Supplemental Table 4B). The canonical astrocyte pathways were most highly enriched in Ast0 and Ast4 (Figure 9D). Ast1 and Ast2 were enriched in pathways related to mitochondrial function, whereas immune activation pathways were enriched in Ast1, Ast2, and Ast5 (Figure 9D). Comparisons between the astrocyte subclusters between *GRN^+/+^* (*Kolf2.1J*) and *GRN^R493X/R493X^* cortical organoids showed that Ast0, Ast1, and Ast2 exhibited downregulation of genes related to synaptic support, but upregulation of genes related to immune activation and inflammation, whereas Ast0, Ast1, Ast2, Ast4, and Ast5 showed mostly downregulation of genes related to protein and lipid metabolism (Figure 9, E and F, and Supplemental Table 4, C–G). Interestingly, comparison of the transcriptomes of *GRN^+/+^* (*Kolf2.1J*) and *GRN^R493X/R493X^* astrocytes revealed genes enriched in mitochondrial ATP synthesis, fatty acid metabolic process, and antimicrobial immune response at 16 and 25 weeks (Figure 9, F and G). Finally, comparison of *GRN^–/–^* astrocyte dataset with *GRN^R493X/R493X^* astrocyte dataset revealed extensive overlaps in the transcriptomic profiles (Figure 9, H and I). Furthermore, *GRN^–/–^* and *GRN^R493X/R493X^* astrocyte clusters shared 100 DEGs, which belonged to GO terms including innate immune response, microtubule cytoskeleton organization, mitotic cell cycle, and negative regulation of neuron development (Figure 9J). Given the significant similarities between the transcriptomic profiles of *GRN^–/–^* and *GRN^R493X/R493X^* astrocytes, we asked whether they also share similar histopathological characteristics. In support of this idea, *GRN^R493X/+^* and *GRN^R493X/R493X^* astrocytes showed significantly more pSMAD3, NF-κB, and pTDP-43, whereas *GRN^R493X/+^* and *GRN^R493X/R493X^* CTIP2^+^ neurons also had increased pTDP-43 expression, albeit not as robust compared with *GRN^R493X/+^* and *GRN^R493X/R493X^* astrocytes (Figure 10, A and B).

## Discussion

TDP-43 proteinopathy is a key diagnostic feature and pathogenic factor in FTLD, AD, and limbic-predominant age-dependent TDP-43 encephalopathy (40, 41). While much attention has focused on the roles of TDP-43 mislocalization in neurons, much less is known about the pathogenesis of TDP-43 proteinopathy in astrocytes and how this impacts astroglial function in neurodegeneration. Here, we leveraged iPSC-derived cortical organoids to show that PGRN deficiency, due to loss-of-function mutation in *GRN* or dominant *GRN^R493X^* mutation, leads to precocious astrogliogenesis, which disrupts the homeostatic interactions between astrocytes, radial glia, and neurons. Mechanistically, PGRN-deficient astrocytes exhibit aberrant TGF-β signaling; promote activation of canonical and noncanonical downstream targets, pSMAD3 and NF-κB, respectively; and activate TTBK2, which can promote phosphorylation of TDP-43. In support of this, blocking TGF-β signaling using TGF-β receptor inhibitors rescues the immune activation and TDP-43 proteinopathy in *GRN^–/–^* astrocytes.

Although TDP-43 proteinopathy has been implicated in promoting neuronal degeneration, emerging evidence indicates that TDP-43 also has critical functions in non-neuronal cells in the central nervous system. Specifically, deleting TDP-43 in microglia promotes amyloid clearance but exacerbates synaptic loss in a mouse AD model (42), whereas TDP-43 directly binds to *SREBF2* mRNA, a transcription regulator of cholesterol metabolism, to regulate cholesterol homeostasis and myelination in the oligodendroglia (43). Moreover, TDP-43 is required for sprouting angiogenesis and blood-brain barrier (BBB) integrity, and endothelium-specific deletion of TDP-43 in postnatal mouse brain disrupts BBB integrity and leads to vascular degeneration and neuroinflammation (44). Finally, conditional expression of a mutant human TDP-43 protein (hTDP-43-DNLS) in hippocampal astrocytes leads to increased expression of interferon-inducible chemokines and chemokine receptor CXCR3 (45). These results support the idea that dysregulation of TDP-43 in astrocytes leads to progressive memory loss and neurodegeneration (46).

Despite these robust results supporting the impacts of mutant TDP-43 in astrocytes, it remains unclear whether TDP-43 accumulation and phosphorylation can spontaneously occur in astrocytes and how these phenotypes are mechanistically linked to astroglial dysfunction and neurodegeneration. Indeed, recent data indicate that the state of TDP-43 phosphorylation can influence its phase separation properties, which may promote its solubility in neurons (47). Our results provide what we believe is the first direct evidence that PGRN deficiency can promote the accumulation of pTDP-43 in astrocytes in *GRN^–/–^* cortical organoids. This phenotype is tightly linked to several detrimental properties of astroglial activation, including the expression of lysosomal protein CTSD, antigen-presenting HLA-D, and immune activator CD44. Finally, using the bioinformatic tool MultiNicheNet, we show that upregulation of TGF-β1 and activation of TGF-β receptors integrin α_v_β_1_ and SDC2 lead to aberrant activation of both canonical and noncanonical TGF-β signaling pathways. These activated astrocytes promote synaptic loss and neuronal stress in *GRN^–/–^* cortical organoids. These results not only provide the first evidence to our knowledge that de novo formation of TDP-43 proteinopathy can occur in a cortical organoid model of FTLD, but they also offer a platform to identify potential therapeutic targets to mitigate astroglial toxicity and TDP-43 proteinopathy.

While this study underscores the advantages of using cortical organoids as a model to investigate the impacts of PGRN deficiency on aberrant astroglial activation and its potential neurotoxicity, it remains unclear how *GRN^–/–^* astrocytes acquire cell-autonomous activation of the TGF-β signaling pathways. It is possible that loss of PGRN may lead to upregulation of TGF-β ligands and receptors in *GRN^–/–^* astrocytes, which could activate TGF-β signaling via an autocrine mechanism. Another potential mechanism is that lysosomal dysfunction in *GRN^–/–^* astrocytes may interfere with the recycling or dampening of activated TGF-β receptors via endolysosomal pathways. Alternatively, defects in extracellular matrix and cell adhesion properties in *GRN^–/–^* astrocytes may promote constitutive activation of TGF-β signaling by converting latent TGF-β into active isoforms (31). Another limitation of this study is that we do not know how activation of TGF-β signaling promotes TDP-43 phosphorylation or whether TDP-43 phosphorylation promotes the activation of *GRN^–/–^* astrocytes. While our results indicate that activation of TDP-43 upstream kinase TTBK2 may contribute to mislocalization of TDP-43 and cytoplasmic accumulation of pTDP-43, the link between TGF-β signaling and TTBK2 activation remains unclear. Future studies will be needed to identify specific mechanisms that promote TDP-43 phosphorylation and astroglial activation in *GRN^–/–^* astrocytes. Finally, while *GRN^–/–^* and *GRN^R493X^* cortical organoids are robust models that capture the multifaceted astroglial phenotypes caused by PGRN haploinsufficiency, these organoids do not recapitulate the cryptic exon retention phenotypes in patients with FTLD-GRN (Figure 6G and Supplemental Figure 9, A and B). This suggests that the cryptic exon retention phenotypes involving neuronal genes may require the prolonged aging process to manifest itself.

## Methods

### Sex as a biological variable

iPSC lines WTC11 and *Kolf2.1J* were derived from male donors. Postmortem human brain tissues were obtained from both male and female patients as previously described (15). Both male and female *Grn^+/+^* and *Grn^–/–^* mice were used.

### iPSC line and expansion culture

Human iPSC lines WTC11 *GRN^+/+^* and WTC11 *GRN^–/–^* (RRID:CVCL_Y803) (48) and *GRN^+/+^* (*Kolf2.1J*, JIPSC003160), *GRN^R493X/+^* (JIPSC003148), and *GRN^R493X/R493X^* (JIPSC003150) (39) were expanded on growth factor reduced (GFR) Matrigel-coated, 6-well plates. Stem cells were thawed in StemFlex Pro Media containing 10 mM ROCK inhibitor Y-27632 (1254; Bio-Techne). After 24 hours, the ROCK inhibitor was removed. Media were then changed every other day and lines passaged when colonies reached about 80% confluence. Stem cells were passaged using ReLeSR (100-0483; STEMCELL Technologies) and residual cells manually lifted with cell lifters. All lines used for this study were between passage 12 and 45. Cells were cultured at 37°C in 5% CO_2_.

### Human brain tissue collection

Deidentified postmortem brain tissues from frontal cortex of patients with FTLD-GRN patients were obtained from the Neurodegenerative Disease Brain Bank at the UCSF as previously described (15). Age-matched control tissues from the same brain regions were obtained from the Autopsy Service in the Department of Pathology at the UCSF. Detailed information regarding age, sex, postmortem interval (PMI), and mutations in the *GRN* gene for FTLD-GRN cases have been previously published (15).

### Mouse brain tissue collection

Mice carrying deletion of the exons 2–13 of the mouse progranulin gene (*Grn^–/–^*) were previously reported by our laboratory (23, 24, 49).

### Cortical organoid differentiation protocol

Cortical organoids were differentiated using the previously published directed differentiation protocol (16). Human iPSC lines were expanded and passaged at least once before being dissociated to single cells using ReLeSR (100-0483; STEMCELL Technologies). After dissociation, cells were reconstituted in neural induction media at 10,000 cells per well in a 96-well, V-bottom, ultralow-adhesion plate. The Glasgow Modified Eagle’s Medium-based neural induction media included 20% Knockout Serum Replacement (10828028; Thermo Fisher Scientific), 1× penicillin-streptomycin (15140122; Thermo Fisher Scientific), 1× nonessential amino acids (11140050; Thermo Fisher Scientific), 0.11 mg/mL sodium pyruvate (11360070; Thermo Fisher Scientific), 0.1 mM β-mercaptoethanol (21985023; Thermo Fisher Scientific), 5 μM TGF-β inhibitor SB431542 (1614; Bio-Techne), and 3 μM WNT inhibitor IWR1-endo (13659; Cayman Chemical). Media were supplemented with 20 μM ROCK inhibitor Y-27632 for the first 6 days. Throughout the entire cortical organoid differentiation protocol, media were changed every other day. At day 18, organoids were transferred into 6-well, ultralow-adhesion plates and moved onto an orbital shaker rotating at 90 rpm. Media were changed to a DMEM/F12 base containing 1× Glutamax, 1× N2 supplement (17502048; Thermo Fisher Scientific), 1× CD Lipid Concentrate (11905031; Thermo Fisher Scientific), and 1× penicillin-streptomycin (15140122; Thermo Fisher Scientific). At day 35, the media were supplemented with 10% fetal bovine serum (SH30071.03; Cytiva), 2.5 μg/mL Amphotericin B (15290018; Thermo Fisher Scientific), 5 μg/mL heparin (H3149; Millipore), and 0.5% GFR Matrigel (356230; Corning). At day 70, media were additionally supplemented with 1× B-27 (17504044; Thermo Fisher Scientific), and the GFR Matrigel concentration was increased to 1%.

### TGF-β inhibition treatment and PGRN replacement protocols

At 20 weeks of differentiation, TGF-β receptor inhibitors LDN193189 (20 nM) (72147; STEMCELL Technologies) and SB431542 (150 nM) (1614; Bio-Techne) were added to the culture media of *GRN^+/+^* and *GRN^–/–^* iPSC-derived cortical organoids. The inhibitors were replenished with every media change throughout the treatment period. At 25 weeks of differentiation, the organoids were collected for immunohistochemical analysis. For progranulin replacement, both *GRN^+/+^* and *GRN^–/–^* cortical organoids were treated with recombinant human progranulin (10 mg/mL) (2420-PG, R&D Systems) from 16 to 25 weeks.

### Immunofluorescence staining

Organoids were fixed in 4% paraformaldehyde for 45 minutes. After 3 washes in PBS, organoids were incubated in 30% sucrose overnight at 4°C. The solution was then replaced with a 1:1 dilution of 50% sucrose and blue OCT and incubated overnight at 4°C. Organoids were then embedded in clear OCT and stored long-term at –80°C. Embedded organoids were cut in 10 μm sections using a Leica cryostat to be used for staining. The staining protocol included antigen retrieval treatment by incubating tissue sections in 10 mM sodium citrate (pH 6.0) at 90°C for 18 minutes. Slides were washed 3 times with PBS-Triton X 0.1% for 5 minutes for permeabilization. Blocking was done in PBS-BSA 3%. DAPI was used for fluorescent nuclear counterstaining.

### Antibodies used in immunofluorescence microscopy

The following primary antibodies were used: anti-CTIP2 clone 25B6 (ab18465; Abcam; RRID:AB_2064130), anti-SATB2 clone SATBA4B10 (ab51502; Abcam; RRID:AB_882455), anti-DCX (AB2253; Millipore; RRID:AB_1586992), anti-Ki-67 clone B56 (556003; BD Biosciences; RRID:AB_396287), anti-NF-κB (8242; Cell Signaling Technology; RRID:AB 10859369), anti-SOX2 (AB5603; Millipore; RRID:AB_2286686), anti-GFAP clone 2.2B10 (13-0300; Invitrogen; RRID:AB_86543), anti-S100B (PA5-24996; Thermo Fisher Scientific; RRID:AB_2542496), anti-SOX9 (AF3075; R&D Systems; RRID:AB_2194160), anti-NES clone 10CS (MAB5326; Millipore; RRID:AB_2251134), anti-TDP-43 (10782-2-AP; Proteintech; RRID:AB_615042), anti-pTDP-43 clone 11-9 (CAC-TIP-PTD-M01A; Cosmo; RRID:AB_3251193), anti-MAP2 (ab5392; Abcam; RRID:AB_2138153), anti-CTSB (AF965; R&D Systems; RRID:AB_2086949), anti-HLA-D clone CR3/43 (ab7856; Abcam; RRID:AB_306142), anti-pSMAD3 clone 16H5L12 (702292; Invitrogen; RRID:AB_2633004), anti-ITGAV (27096-1-AP; Proteintech; RRID:AB_2880753), anti-CytoC clone 6H2.B4 (556432; BD Biosciences; RRID:AB_396416), anti-GORASP2 (10598-1-AP; Proteintech; RRID:AB_2113473), anti-cCas3 (9664; Cell Signaling Technology; RRID:AB_2070042), anti-PSD95 (ab13552; Abcam; RRID:AB_300453), and anti-BSN (141-002; Synaptic Systems; RRID:AB_887698).

The following secondary antibodies were used: Donkey anti-rat AlexaFluor 488 (A21208; Invitrogen; RRID:AB_141709), Donkey anti-rat AlexaFluor 647 (ab150155; Abcam; RRID:AB_2813835), Donkey anti-mouse AlexaFluor 568 (A10037; Invitrogen; RRID:AB_11180865), Donkey anti-mouse AlexaFluor 647 (A31571; Invitrogen; RRID:AB_162542), Donkey anti-guinea pig AlexaFluor 488 (706-545-148; Jackson Immunoresearch; RRID:AB_2340472), Donkey anti-rabbit AlexaFluor 488 (A21206; Invitrogen; RRID:AB_2535792), Donkey anti-rabbit AlexaFluor 647 (A31573; Invitrogen; RRID:AB_2536183), Donkey anti-rat AlexaFluor 405 (ab175670; Abcam; RRID:AB_3099480), Donkey anti-goat AlexaFluor 568 (A11057; Invitrogen; RRID:AB_2534104), Donkey anti-goat AlexaFluor 647 (A21447; Invitrogen; RRID:AB_2535864), Donkey anti-chicken AlexaFluor 488 (703-545-155; Jackson Immunoresearch; RRID:AB_2340375), Donkey anti-chicken AlexaFluor 633 (20168; Biotium; RRID:AB_10853143), Donkey anti-goat AlexaFluor 488 (A11055; Invitrogen; RRID:AB_2534102), and Donkey anti-mouse AlexaFluor 488 (A21202; Invitrogen; RRID:AB_141607).

### Image acquisition and quantification

Images were taken on a Nikon C2 confocal microscope using the NIS-Elements AR 5.30.02 software (RRID:SCR_014329). The 10× images were taken on a single plane and used 3×3 stitching. *Z*-stacks were taken at 60× with zoom 1 images using 10 steps of 1 μm and zoom 2 and 3 images using 10 steps of 0.5 μm. Quantifications in were done on Fiji (RRID:SCR_002285) using the Cell Counter macro on max-projection images.

### Western blots

Samples containing 40 mg proteins were resolved on NuPAGE Novex 4%–12% Bis-Tris gradient gel in MOPS SDS Running Buffer at 200 V constant for 50 minutes. Proteins were transferred onto polyvinylidene fluoride (PVDF) membranes (Invitrogen) using the XCell SureLock Mini-Cell system (Invitrogen). Wet transfer was performed at 300 mA for 2 hours at 4°C. Blots were then blocked with 5% skimmed milk powder in Tris-buffered saline (TBS, pH:7.5) containing 0.1% Tween-20 (TBS-T) for 2 hours at room temperature (RT). Primary antibodies were incubated overnight at 4°C before the membranes were washed with 1× TBS-T solution 5 times for 7 minutes the next day. The secondary antibodies were incubated with the membranes on a shaker for 2 hours at RT. PVDF membrane was again washed 5 times with TBS-T for 7 minutes each. The membrane was completely incubated with the Clarity Western ECL Substrate (Bio-Rad) for 3 minutes. Intensity of immunoblot bands was quantified using ImageJ software (NIH). The following antibodies were used in Western blots: anti-GAPDH (T0004; Affinity Biosciences; RRID: AB_2833041), anti-CD44 (HPA005785; Sigma; RRID: AB_1078467), anti-pTDP-43 (829901; BioLegend; RRID: AB_2564934), anti-NF-κB (3033s; Cell Signaling Technology; RRID: N/A), anti-pNF-κB (8242s; Cell Signaling Technology; RRID: N/A), anti-pSMAD3 (702292; Thermo Scientific; RRID:AB_2633004), anti-TTBK2 (15072-1-AP; Proteintech; RRID: AB_2211507), anti-TTBK1 (sc-374600; Santa Cruz Biotechnology; RRID: AB_10988089), and anti-CDC7 (3603; Cell Signaling Technology; RRID: AB_2276095). The following secondary antibodies were used in Western blots: Peroxidase (HRP) Horse anti-Mouse IgG Horse (7076P2; Cell Signaling Technology; RRID: AB_330924), Peroxidase (HRP) Goat anti-Rabbit IgG (7074P2; Cell Signaling Technology; RRID: AB_2099233), and Peroxidase (HRP) Donkey anti-Rat IgG (712-035-150; Jackson ImmunoResearch; RRID: AB_2340638).

### Transcriptomics

#### scRNA-seq pipeline.

To prepare the cell suspension, organoids were incubated in a solution of Earle’s Balanced Salt Solution, 20 U/mL papain, 1 mM l-cysteine, 0.5 mM EDTA, and 2,000 U/mL deoxyribonuclease for 45 minutes at 37°C (Worthington Biochemical LK003150). Every 10 minutes, gentle pipetting was used to further break down the organoids. At 45 minutes, medium was added to stop the reaction, and the tubes were spun down at 300*g* for 5 minutes. The media were replaced with cold 0.5% BSA in PBS. The samples were filtered once through FACS filter tubes. Additional 0.5% BSA in PBS was used to dilute the samples to reach 1,000 cells/μL. Cells were processed using the Chromium Next GEM Single Cell 3′ Gene Expression kit (version 3.1) according to the manufacturer’s instructions. In short, 16,500 cells per sample were loaded onto the Next GEM Single Cell Chip G to maximize cells captured while minimizing doublets (targeting 10,000 cells per sample). Using a Chromium Controller, gel bead-in emulsions (GEMs) were created and thereafter processed to isolate and amplify cDNA and construct libraries. Quality and concentration of cDNA and libraries were evaluated on the Agilent 2100 Bioanalyzer (RRID:SCR_019715). Libraries were sequenced on an Illumina NovaSeq 6000 machine (RRID:SCR_016387) (average depth 500 million reads/sample) at the UCSF Genomics Core Facility. BCL base call files were converted to FASTQ files using bcl2fastq Conversion software (version 2.20; RRID:SCR_015058). Using Cell Ranger software suite (version 7.1.0; RRID:SCR_017344) (10x Genomics Cloud Analysis), FASTQ files were aligned to reference genome Human (GRCh38) v3.0.0, and gene-barcode count matrices were generated for all demultiplexed samples.

#### Quality control, normalization, and dimensional reduction.

Sequencing analysis was conducted using the Seurat pipeline (version 5.1.0; RRID:SCR_016341) on R (version 4.3.2; RRID:SCR_001905). Sample matrices were converted into Seurat objects and merged. For quality control on the WTC11 organoids, cells were removed if they did not have percent mitochondria < 15%, percent ribosome < 35%, counts ≤ 30,000, and unique molecular identifiers (UMIs) ≤ 7,000. For quality control on the *Kolf2.1J* organoids, cells were removed if they did not have percent mitochondria < 15%, percent ribosome < 40%, counts ≤ 10,000, and UMIs ≤ 4000. These were each optimized to reach an *r* value close to 1.00 on the counts versus UMI plot. The Seurat object was log-normalized and the top 2,000 variable features were identified. After scaling the data, linear dimensional reduction was performed by principal component analysis of variable features using the top 14 principal components for WTC11 organoids and top 20 principal components for *Kolf2.1J* organoids, as determined based on the inflection point of an Elbow plot. The data were also normalized using the function *SCTransform*. DoubletFinder (version 2.0.4; RRID:SCR_018771) was used to find and remove doublets. Harmony (version 1.2.0; RRID:SCR_022206) was used for batch correction.

#### Clustering and annotation.

Clustering was calculated using the functions *RunUMAP*, *FindNeighbors*, and *FindClusters*, with a range in resolution between 0.20 and 1. A final resolution of 0.25 was decided upon for WTC11 organoids and a final resolution of 0.20 was decided upon for *Kolf2.1J* organoids. Clusters were annotated as major cell types based on series of canonical markers as queried by *FindMarkers*, *DotPlot*, and *FeaturePlot*. The scvelo package (version 0.3.1; RRID:SCR_018168) on Python (version 3.10.10; RRID:SCR_008394) was used to calculate the pseudotime analysis and plot the RNA velocity.

#### Comparison with Bhaduri et al., 2020.

Cells were compared with a previously published cortical organoid dataset described in Bhaduri et al. (NCBI GEO: GSE132672), using the label transfer pipeline described for Seurat v5 (*FindTransferAnchors* and *MapQuery* functions). These analyses projected cells onto the UMAP space of the Bhaduri et al., 2020, cortical organoid dataset and transferred cell types from the Bhaduri et al., 2020, cortical organoid dataset.

#### Measurement of region-specific gene signatures.

Brain region-specific transcriptomic signatures were derived from Bhaduri et al. (20). Signatures for the ganglionic eminence, striatum, thalamus, and cerebellum were identified by isolating the top 10 markers for each of these regions, agnostic of cell type. Signatures for individual cortical areas were derived for each area by first isolating the top areal markers across all cell types identified in Bhaduri et al. (20), then filtering these based on their specificity to and enrichment in the indicated area (i.e., markers with a Gene Score > 1). The activity of these signatures in each cell was evaluated using a module activity score as described in Nano et al. (50), which calculates the average normalized counts per million detected for each gene in the indicated signature.

#### Astroglia subclustering.

The glioblast and astrocyte clusters from the main UMAP were subset and combined for reclustering. To ensure compatibility with the Sadick dataset for future analysis, reclustering of our astroglia followed their protocol (25). The Seurat object was converted to a SingleCellExperiment (SCE). It was split by sample, normalized, scaled, and reintegrated. Clustering was calculated using the functions *RunPCA*, *RunTSNE*, *RunUMAP*, *FindNeighbors*, and *FindClusters*, with a range in resolution between 0.05 and 0.20. A final resolution of 0.08 was decided upon for WTC11 organoids, and a final resolution of 0.10 was decided upon for *Kolf2.1J* organoids. DEG and pathway analysis was conducted in 3 ways using org.Hs.eg.db (version 3.18.0; RRID:SCR_024739) and clusterProfiler (version 4.10.1; RRID:SCR_016884): (a) comparing genotypes within the entire astroglia object split by age, (b) comparing genotypes within each subcluster, and (c) comparing each subcluster to each other. The scvelo package (version 0.3.1; RRID:SCR_018168) on Python (version 3.10.10; RRID:SCR_008394) was used to calculate the pseudotime analysis and plot the RNA velocity.

#### Reanalysis of other datasets.

First, we reproduced the analysis from the Sadick paper, starting with the raw samples and following their published code (GEO: GSE167494). However, instead of using tNSE like the paper, we projected onto a UMAP. Next, FindTransferAnchors was used to find anchors for integration between the Sadick dataset and this paper’s dataset. Then, we projected the query (this paper) onto the reference UMAP (Sadick). Finally, we conducted DEG analysis on the 2 datasets, identified the DEGs in common, and conducted pathway enrichment analysis on those DEGs. The same process was used for each of the projection combinations shown.

#### Cell-cell communication analysis.

First, the cell type categories were simplified to combine the numbered clusters under its parent category, and the smaller clusters were excluded. The resulting categories included radial glia, inhibitory neurons, CTIP2^+^ neurons, CTIP2^+^SATB2^+^ neurons, SATB2^+^ neurons, and astrocytes. The MultiNicheNet package (version 2.0.0; RRID:SCR_025903) was used to conduct the cell communication network analysis with the Seurat object converted to a SCE (29). First, we did cell type filtering, controlling for batch and genotype, to ensure that there were sufficient counts present in each cell type for further analysis. CTIP2^+^ neurons and CTIP2^+^SATB2^+^ neurons were removed at this stage due to insufficient cell count in each genotype. Second, the genes were filtered to determine which were sufficiently expressed in each cell type with a sample proportion cutoff of 0.50 and a minimum expression cutoff of 0.05. Third, we conducted a pseudobulk expression analysis for each expressed gene in each present cell type. Fourth, DEG analysis was performed between *GRN^+/+^* and *GRN^–/–^*. Fifth, we used the output to predict the activity of ligands in receiver cell types and infer their potential target genes. Finally, ligand-receptor pairs were ranked by the aggregated prioritization score. This score equally accounts for ligand-receptor expression, genotype specific upregulated ligand-receptor pairs, and ligand-receptor pairs that have high predicted impact on gene expression.

### Statistics

#### Immunostainings quantification.

The exact numbers of samples and images used are indicated in the figure legends. Statistical analyses were done using GraphPad Prism (version 10.3.0; RRID:SCR_002798). For comparisons between 2 groups, if normally distributed, 2-tailed unpaired Student’s *t* tests were performed. If non-normally distributed, Mann-Whitney *U* tests were performed. *P* < 0.05 was considered significant. For comparisons of more than 2 groups, 2-way ANOVA with Tukey’s multiple comparisons test were performed.

#### Transcriptomic analysis.

The exact numbers of biological and experimental replicates are indicated in the figure legends. Differential gene expression statistics were performed using MAST statistical tests (version 1.28.0; RRID:SCR_016340). GO term *P* values and G-scores were obtained directly from the clusterProfiler (version 4.10.1; RRID:SCR_016884) analysis results. The Venn diagram statistical comparison was assessed using a hypergeometric test.

### Study approval

Brain tissue samples were collected after written informed consent was obtained from the patients or their families, in accordance with guidelines put forth in the Declaration of Helsinki. Autopsy consent and all protocols were approved by the Human Gamete, Embryo, and Stem Cell Research Committee and the Institutional Review Board at UCSF. All mouse experiments were conducted in accordance with the UCSF Institutional Animal Care and Use Committee guidelines (IACUC Protocol AN169548).

### Data availability

scRNA-seq data have been deposited at GEO, and the accession number is GSE282668. All original code has been deposited at Zenodo (https://doi.org/10.5281/zenodo.20709533) and is publicly available as of the date of publication. Any additional information required to reanalyze the data reported in this paper is available from the corresponding author upon request. All raw data values are included in the Supporting Data Values file.

## Author contributions

ACR, XYT, EM, and EJH conceived the project and designed the experiments. ACR, XYT, and EM prepared cortical organoids. ACR and XYT performed immunohistochemistry. ACR and EM performed 10X Genomics scRNA-seq. ACR conducted bioinformatic analyses, with inputs from RL. ACR and XYT characterized TGF-β signaling and pTDP-43 phenotypes. PLRN compared scRNA-seq data from organoids with region-specific gene expression signatures from prenatal human brain, with supervision from AB. MJM, BB, and CML assisted with immunohistochemical characterizations of cortical organoids, with supervision from ACR. JZ and WB performed IEM for pTDP-43. JP, TM, and ARK contributed reagents and expertise. ACR, XYT, and EJH wrote the manuscript with inputs from MJM, PRN, RL, BB, CML, JP, JZ, WB, TM, ARK, AB, and EM.

## Conflict of interest

ARK is a founding member and scientific advisor of Neurona Therapeutics.

## Funding support

This work is the result of NIH funding, in whole or in part, and is subject to the NIH Public Access Policy. Through acceptance of this federal funding, the NIH has been given a right to make the work publicly available in PubMed Central.

- NIH grant R01MH132689 (to AB).
- NIH grants R01 AG057462, R01 AG068290, and R01 NS128908 (to EJH).
- VA BLR&D Merit Review Award I01 BX001108 (to EJH).
- NIGMS IMSD fellowship (ACR).

## Supplementary Material

Supplemental data

Unedited blot and gel images

Supplemental table 1

Supplemental table 2

Supplemental table 3

Supplemental table 4

Supporting data values

## Version 1 06/16/2026

In-Press Preview

## Version 2 07/15/2026

Electronic publication
