# Supplementary material for: TGF-β signaling promotes astroglial activation and TDP-43 proteinopathy in organoid models of frontotemporal lobar degeneration
Source: J Clin Invest. 2026 Jun 16;136(14):e190035. doi: 10.1172/JCI190035 (PMC13367973; doi:10.1172/JCI190035)
Supplement: Supplemental data [file jci-136-190035-s093.pdf]

Supplementary Information

TGFβ signaling promotes astroglial immune activation and TDP-43 proteinopathy in an organoid model of FTLD

Arren C. Ramsey, Xiao-Yan Tang, Magdalena J. Macias, Patricia R. Nano, Rufei Lu, Brian Benito, Cameron M. Lau, Jisu Park, Jiasheng Zhang, Wandy Beatty, Tanzila Mukhtar, Arnold R. Kriegstein, Aparna Bhaduri, Elise Marsan, Eric J. Huang

Content: 11 Supplementary Figures & Legends

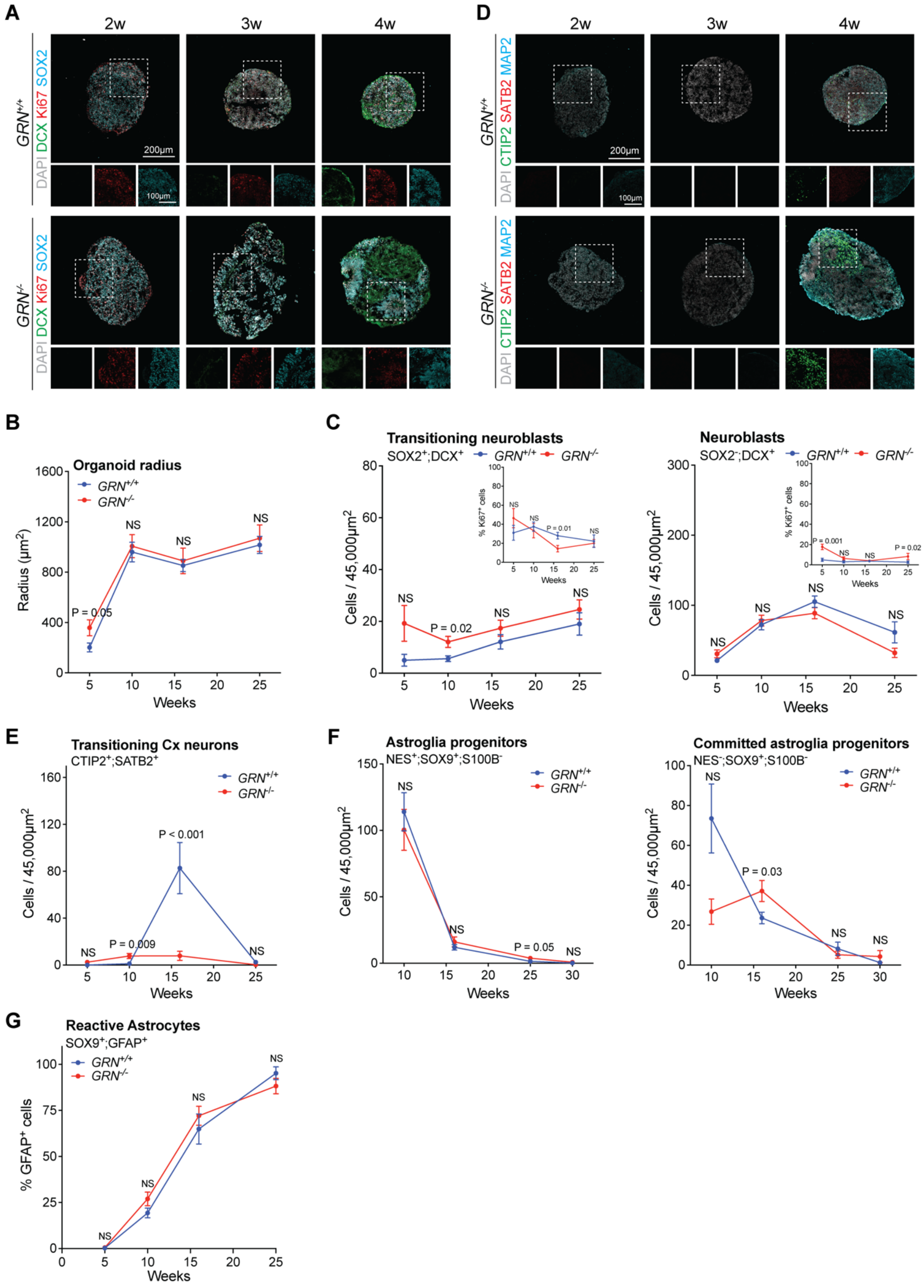
A
2w
3w
4w
GRN+/+
DAPI DCX Ki67 SOX2
200µm
100µm
GRN-/-
D
CTIP2 SATB2 MAP2
B
Organoid radius
Radius (µm2)
Weeks
P = 0.05
NS
C
Transitioning neuroblasts
SOX2+;DCX+
Cells / 45,000µm2
% Ki67+ cells
P = 0.02
P = 0.01
Neuroblasts
SOX2-;DCX+
P = 0.001
P = 0.02
E
Transitioning Cx neurons
CTIP2+;SATB2+
P < 0.001
P = 0.009
F
Astroglia progenitors
NES+;SOX9+;S100B-
P = 0.05
Committed astroglia progenitors
NES-;SOX9+;S100B-
P = 0.03
G
Reactive Astrocytes
SOX9+;GFAP+
% GFAP+ cells

Supplementary Figure S1. Related to Figure 1. Cell type-specific developmental trajectories of *GRN*$^{+/+}$ and *GRN*$^{-/-}$ cortical organoids. (A) Confocal images of immunostains for DCX, Ki-67, SOX2 in *GRN*$^{+/+}$ and *GRN*$^{-/-}$ iPSC-derived cortical organoids at early developmental time points (2w, 3w, and 4w). **(B)** Quantification of organoid radius at different developmental time points in *GRN*$^{+/+}$ (blue) and *GRN*$^{-/-}$ (red) (5w, 10w, 16w, and 25w). Organoids (n = 9) from 3 independent biological replicate experiments were analyzed per time point for each genotype. **(C)** Quantification of the density of SOX2^{+};DCX^{+} transitioning neuroblasts and SOX2^{-};DCX^{+} neuroblasts at different developmental time points in *GRN*$^{+/+}$ (blue) and *GRN*$^{-/-}$ (red). Inset shows the percentage of Ki67^{+} cells among transitioning neuroblasts and neuroblasts over time. Organoids (n = 9) from 3 independent biological replicates were analyzed per time point for each genotype. **(D)** Confocal images of immunostains for CTIP2, SATB2, MAP2 in *GRN*$^{+/+}$ and *GRN*$^{-/-}$ iPSC-derived cortical organoids at early developmental time points (2w, 3w, and 4w). **(E)** Quantification of the density of CTIP2^{+};SATB2^{+} transitioning cortical neurons at different developmental time points in *GRN*$^{+/+}$ (blue) and *GRN*$^{-/-}$ (red). Organoids (n = 9) from 3 independent biological replicate experiments were analyzed per time point for each genotype. **(F)** Quantification of the density of NES^{+};SOX9^{+};S100β^{-} astroglia progenitors and NES^{-};SOX9^{+};S100β^{-} committed astroglia progenitors at different developmental time points in *GRN*$^{+/+}$ (blue) and *GRN*$^{-/-}$ (red). Organoids (n = 9) from 3 independent biological replicate experiments were analyzed per time point for each genotype. **(G)** Quantification of the percentage of GFAP^{+};SOX9^{+} cells out of all SOX9^{+} cells at different developmental time points in *GRN*$^{+/+}$ (blue) and *GRN*$^{-/-}$ (red). Organoids (n = 9) from 3 independent biological replicate experiments were analyzed per time point for each genotype. All data represent mean ± SEM.

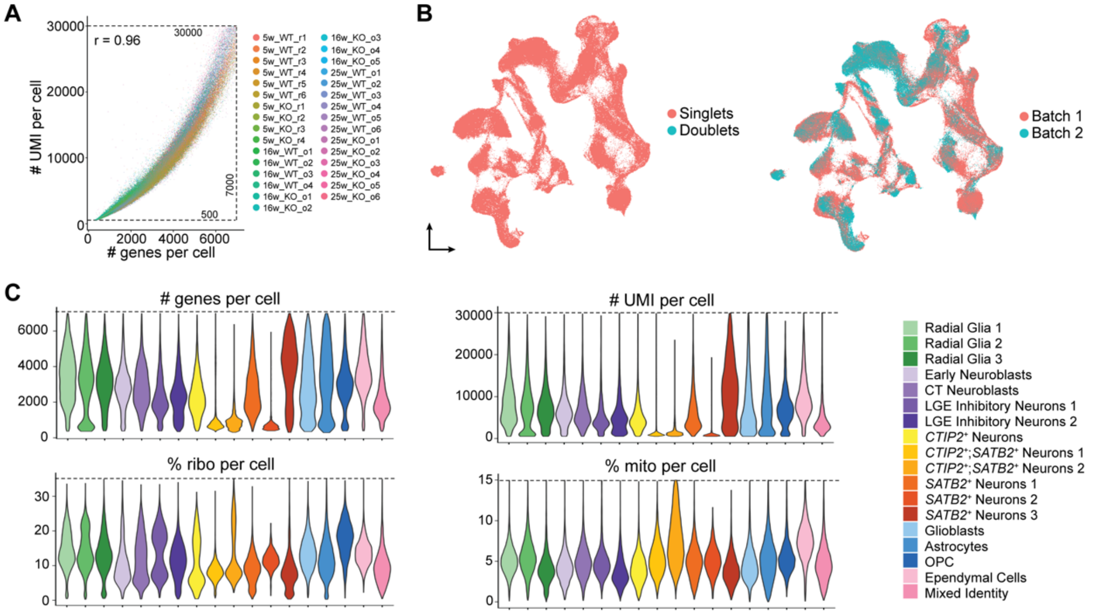

D

	5w *GRN*-/-	5w *GRN*+/+	16w *GRN*-/-	16w *GRN*+/+	25w *GRN*-/-	25w *GRN*+/+
Cells	43,918	54,465	36,539	35,564	29,757	29,656
Biological Replicates	4	6	5	4	6	6
Experimental Replicates	2	2	2	2	2	2

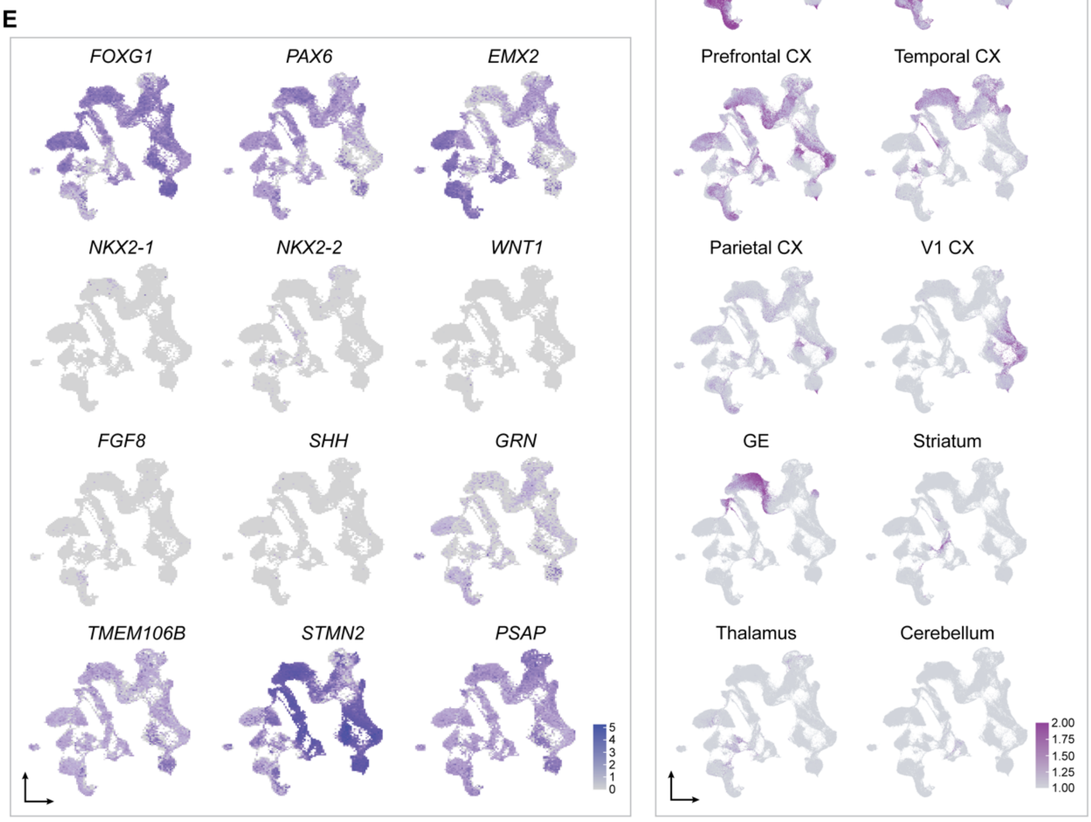

Supplementary Figure S2. Relative to Figure 2. Single-cell transcriptomic analyses of *GRN*$^{+/+}$ and *GRN*$^{-/-}$ cortical organoids. (A) Scatterplot grouped by sample showing the correlation between the number of unique molecular identifiers (UMI) per cell and the number of genes per cell. **(B)** UMAPs grouped by singlet vs doublet (right) and experimental batch (left). **(C)** Violin plots grouped by cluster showing the number of genes per cell (top left), number of UMI per cell (top right), percentage of ribosomal genes per cell (bottom left), and percentage of mitochondrial genes per cell (bottom right). **(D)** Table showing the number of cells, biological replicates, and experimental replicates per age and genotype. **(E)** Feature plots of regional identity markers and FTLD-specific markers. **(F)** Feature plots of region-specific gene signature as calculated in STAR Methods.

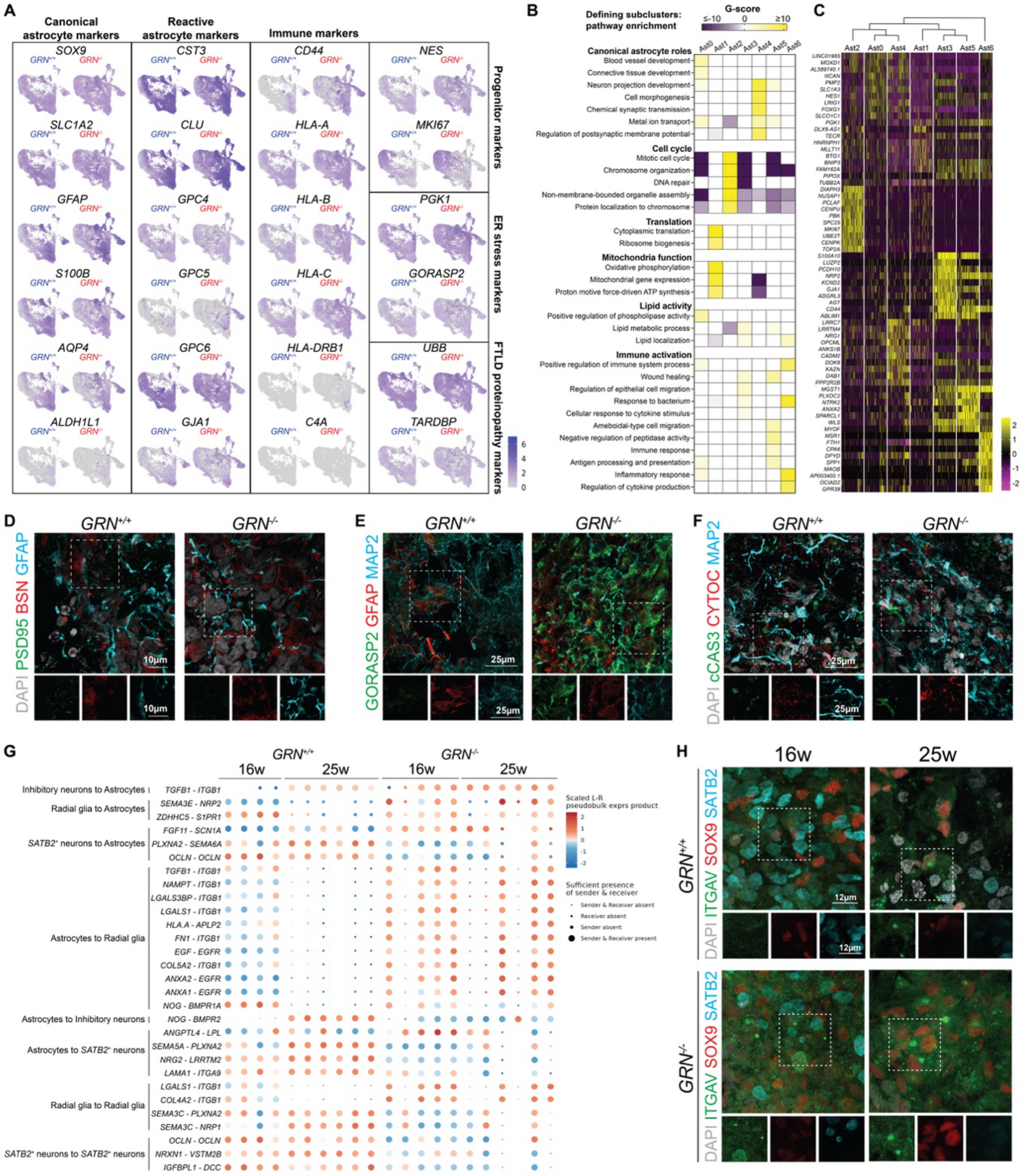

Supplementary Figure S3. Related to Figure 3. Transcriptomic profiles of astrocyte subclusters, MultiNicheNet analyses of the signaling mechanisms mediating cell-cell communications, and histopathological characterizations of synaptic loss, cellular stress, and cell death in *GRN*$^{+/+}$ and *GRN*$^{-/-}$ cortical organoids. (A) Feature plots of canonical astrocyte markers, reactive astrocyte markers, immune markers, progenitor markers, ER stress markers, and FTLD

proteinopathy markers. **(B)** Heatmap of top up- and down-regulated GO terms in each astroglia cluster. G-score refers to avgFC*-log(adj_p_val). **(C)** Heatmap of top DEGs in each astroglia cluster. Clusters are sorted by hierarchal similarity (top). **(D)** Confocal images showing reduced synaptic markers bassoon (BSN) and PSD95 in $GRN^{-/-}$ cortical organoids at 25 weeks. **(E)** Confocal images showing increased ER stress marker GORASP2 in $GRN^{-/-}$ cortical organoids at 25 weeks. **(F)** Confocal images showing diffuse cytochrome C (CYTOC) and increased cleaved caspase 3 (cCAS3) in MAP2^{+} neurons $GRN^{-/-}$ cortical organoids at 25 weeks. **(G)** Heatmap of ligand-receptor pairs of various cell type combinations split by age and genotype. **(H)** Confocal images of immunostains for ITGAV, SOX9, and SATB2 in $GRN^{-/-}$ and $GRN^{+/+}$ iPSC-derived cortical organoids at 16 and 25 weeks.

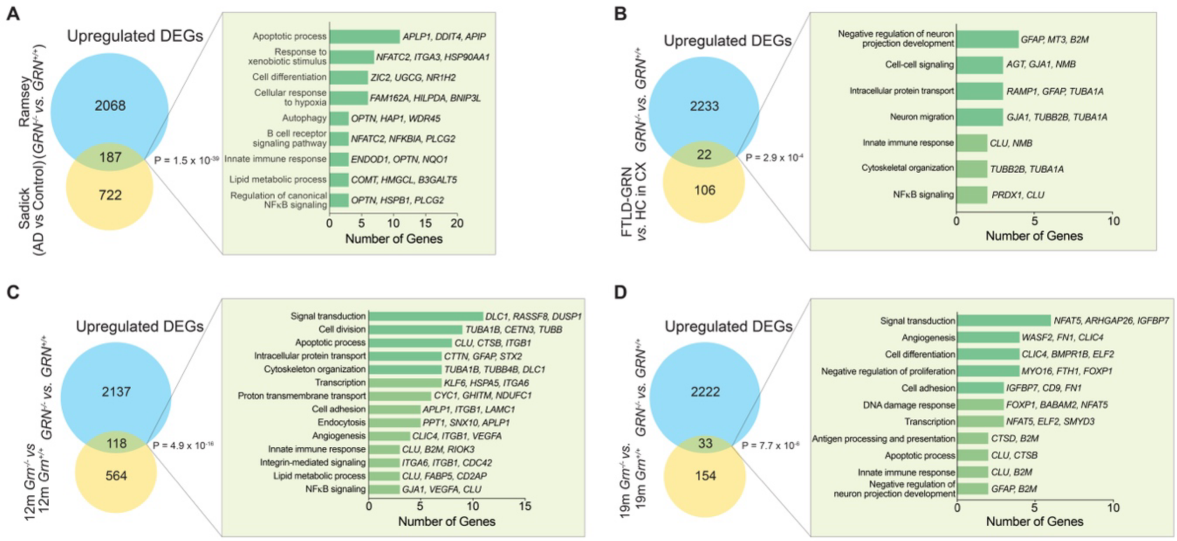

Supplementary Figure S4. Related to Figure 3. Comparative analyses of *GRN*$^{-/-}$ astrocyte transcriptomic profiles. (A-D) Venn diagrams showing the overlap of the up-regulated DEGs between Ramsey (*GRN*$^{-/-}$ vs *GRN*$^{+/+}$) and Sadick (Ctrl vs AD) datasets **(A)**, overlap of the up-regulated DEGs between *GRN*$^{-/-}$ vs *GRN*$^{+/+}$ and FTLD vs HC in CX datasets **(B)**, overlap of the up-regulated DEGs between *GRN*$^{-/-}$ vs *GRN*$^{+/+}$ and 12-month *Grn*$^{-/-}$ vs *Grn*$^{+/+}$ datasets **(C)**, overlap of the up-regulated DEGs between *GRN*$^{-/-}$ vs *GRN*$^{+/+}$ and 19-month *Grn*$^{-/-}$ vs *Grn*$^{+/+}$ datasets **(D)**. Statistics used hypergeometric test. Right panels represent bar graphs showing GO-terms based on the overlapping DEG lists (left).

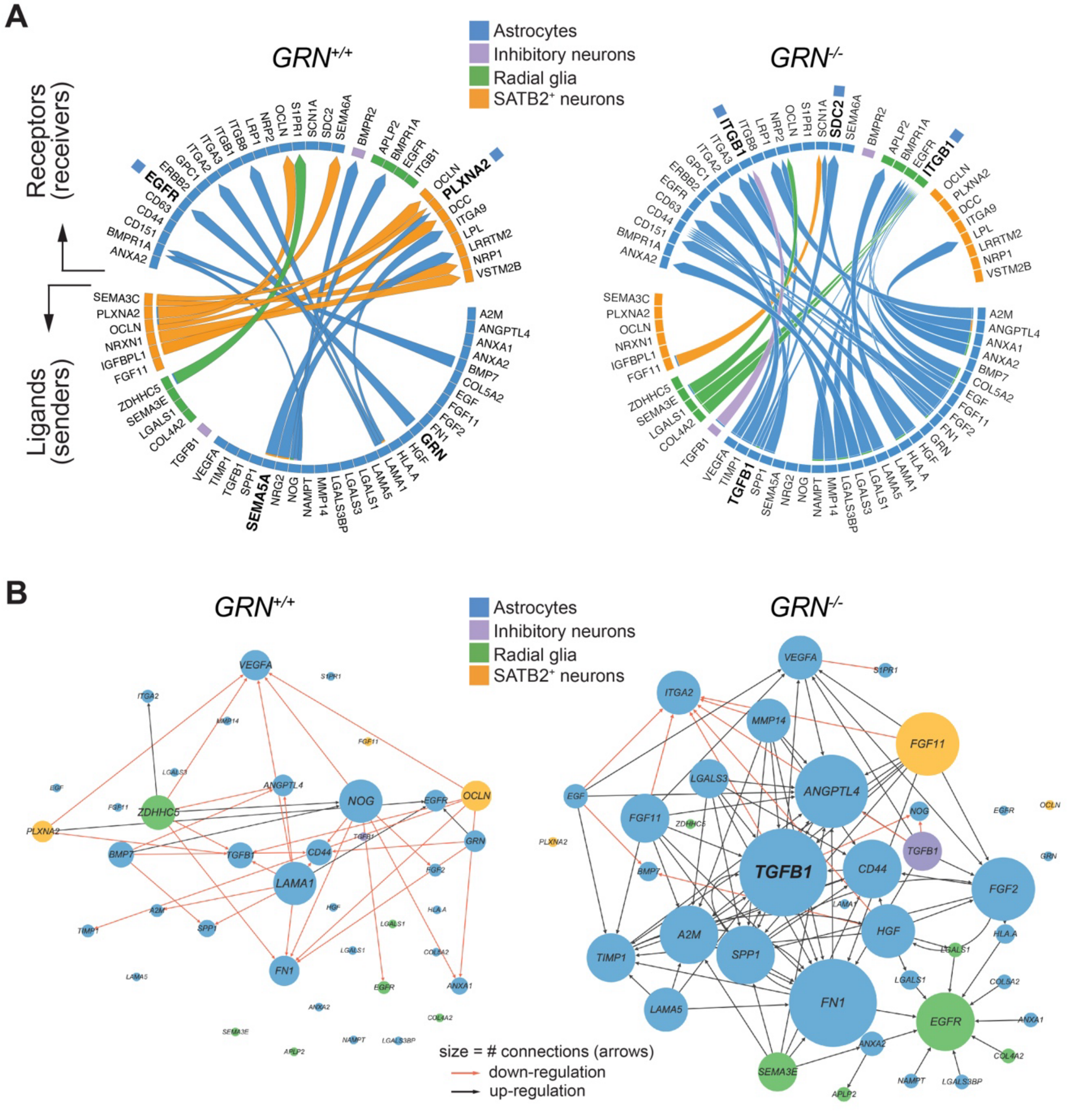

Supplementary Figure S5. Related to Figure 4. MultiNicheNet analyses of the signaling mechanisms mediating cell-cell communications in *GRN*$^{+/+}$ and *GRN*$^{-/-}$ cortical organoids. (A) Ligand-receptor pairs for the cell-cell communication network in *GRN*$^{+/+}$ and *GRN*$^{-/-}$ organoids. Bottom lists ligands expressed in sender cell type and top lists receptors expressed in receiver cell type. **(B)** Cell-cell communication network in *GRN*$^{+/+}$ and *GRN*$^{-/-}$ organoids. Size of dot is proportional to the number of connections, and color of line represents directionality of relationship (black = up-regulation, red = down-regulation).

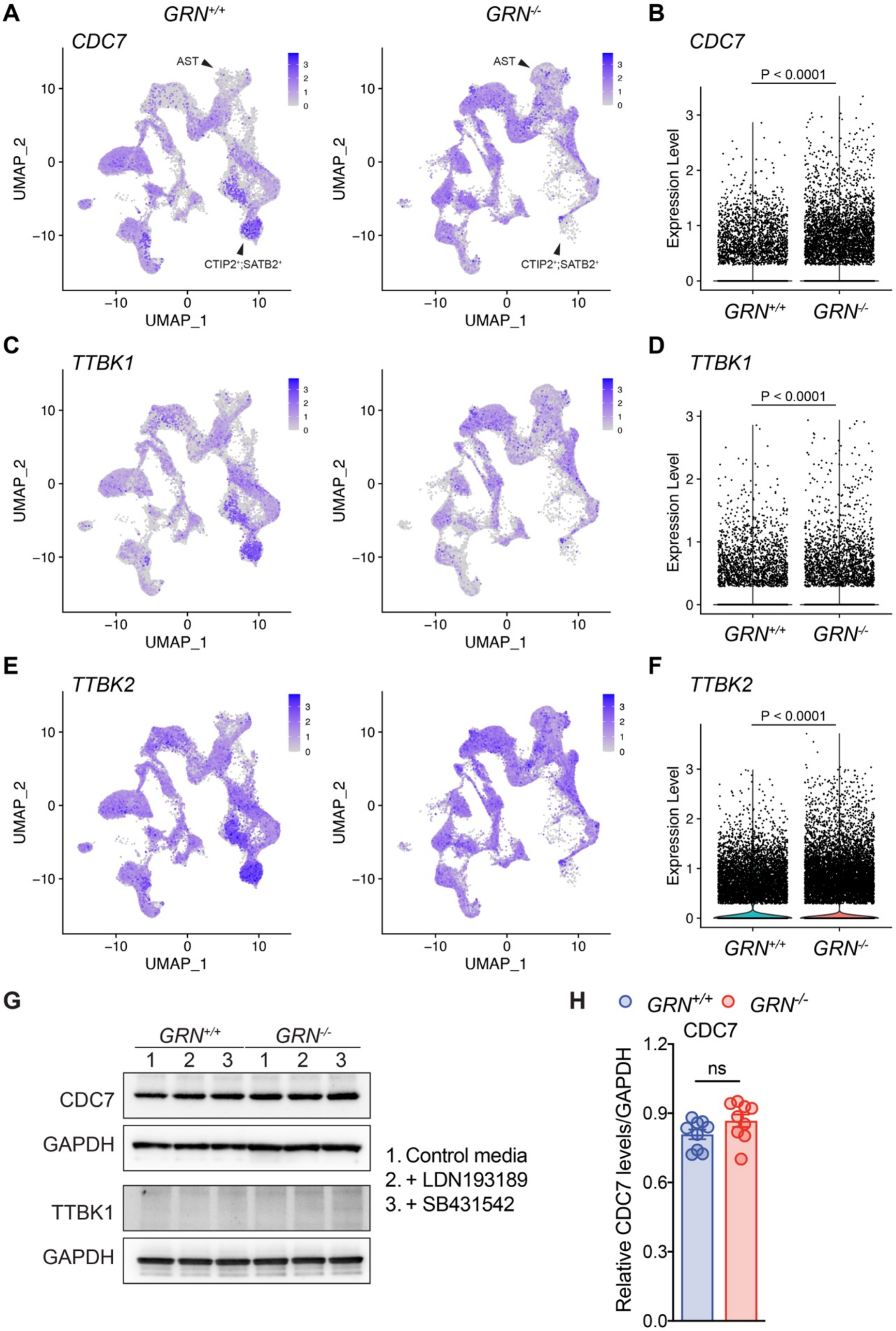

A
GRN+/+
GRN-/-
CDC7
AST
CTIP2+;SATB2+
UMAP_1
UMAP_2
B
CDC7
P < 0.0001
Expression Level
C
TTBK1
D
TTBK1
P < 0.0001
E
TTBK2
F
TTBK2
P < 0.0001
G
CDC7
GAPDH
TTBK1
1. Control media
2. + LDN193189
3. + SB431542
H
CDC7
ns
Relative CDC7 levels/GAPDH

Supplementary Figure S6. Related to Figure 4. Expression of TDP-43 kinases via scRNA-seq and western blots. (A, C, E) UMAPs showing the expression of *CDC7* (**A**), *TTBK1* (**C**), and *TTBK2* (**E**) in $GRN^{+/+}$ and $GRN^{-/-}$ cortical organoids. **(B, D, F)** Violin plots of *CDC7* (**B**), *TTBK1* (**D**), and *TTBK2* (**F**) transcript levels in astrocyte clusters in $GRN^{+/+}$ and $GRN^{-/-}$ cortical organoids. Statistics used Student's *t* test. **(G-H)** Western blots and quantification showing that treatment with TGFβ receptor inhibitors, LDN193189 (20 nM, lane 2) or SB431542 (150 nM, lane 3) had no effects on the relative abundance of CDC7 in $GRN^{+/+}$ and $GRN^{-/-}$ cortical organoids. In contrast to CDC7, western blots failed to detect TTBK1 in the same cell lysates prepared from $GRN^{+/+}$ and $GRN^{-/-}$ cortical organoids. Statistics used Student's *t* test.

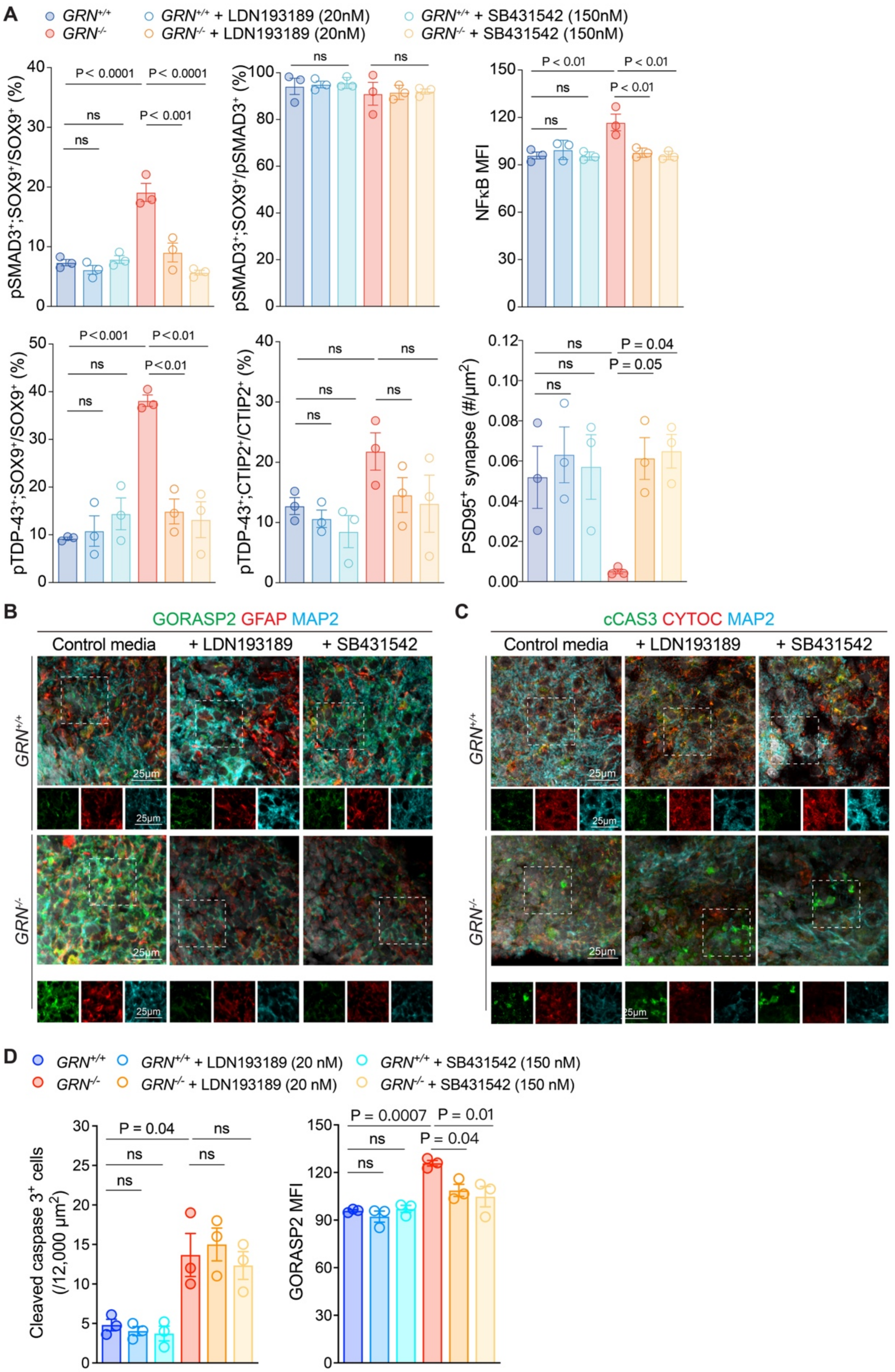
A
GRN+/+
GRN-/-
GRN+/+ + LDN193189 (20nM)
GRN-/- + LDN193189 (20nM)
GRN+/+ + SB431542 (150nM)
GRN-/- + SB431542 (150nM)
pSMAD3+;SOX9+/SOX9+ (%)
pSMAD3+;SOX9+/pSMAD3+ (%)
NFκB MFI
pTDP-43+;SOX9+/SOX9+ (%)
pTDP-43+;CTIP2+/CTIP2+ (%)
PSD95+ synapse (#/µm2)
P< 0.0001
P< 0.001
P< 0.01
P<0.001
P<0.01
P = 0.04
P = 0.05
ns
B
GORASP2 GFAP MAP2
C
cCAS3 CYTOC MAP2
Control media
+ LDN193189
+ SB431542
GRN+/+
GRN-/-
25µm
D
GRN+/+
GRN-/-
GRN+/+ + LDN193189 (20 nM)
GRN-/- + LDN193189 (20 nM)
GRN+/+ + SB431542 (150 nM)
GRN-/- + SB431542 (150 nM)
Cleaved caspase 3+ cells (/12,000 µm2)
GORASP2 MFI
P = 0.04
P = 0.0007
P = 0.01
ns

Supplementary Figure S7. Related to Figure 5. Effects of TGFβ receptor inhibitors in *GRN*$^{-/-}$ cortical organoids. (A) Quantification of the proportion of pSMAD3$^+$;SOX9$^+$ cells out of SOX9$^+$ cells (top row, left graph); pSMAD3$^+$;SOX9$^+$ cells out of pSMAD3$^+$ cells (top row, middle graph); NFκB MFI (top row, right graph); pTDP43$^+$;SOX9$^+$ cells out of SOX9$^+$ cells (bottom row, left graph); pTDP43$^+$;CTIP2$^+$ cells out of CTIP2$^+$ cells (bottom row, middle graph); and PSD95$^+$ synapse/μm^2 (bottom row, right graph) in *GRN*$^{+/+}$ and *GRN*$^{-/-}$ cortical organoids treated with LDN193189 or SB431542 from 20 to 25 weeks. **(B-C)** Confocal images of GORASP2, GFAP, and MAP2 **(B)** and cleaved caspase 3, Cytochrome C (CYTOC), and MAP2 **(C)** in *GRN*$^{+/+}$ and *GRN*$^{-/-}$ cortical organoids. **(D)** Quantification of cleaved caspase 3$^+$ cells and GORASP2$^+$ signaling intensity in GFAP$^+$ astrocytes in *GRN*$^{+/+}$ and *GRN*$^{-/-}$ cortical organoids. Organoids (n = 3) from 1 biological replicate. Data represent mean ± SEM. Statistics used two-way ANOVA with multiple comparisons.

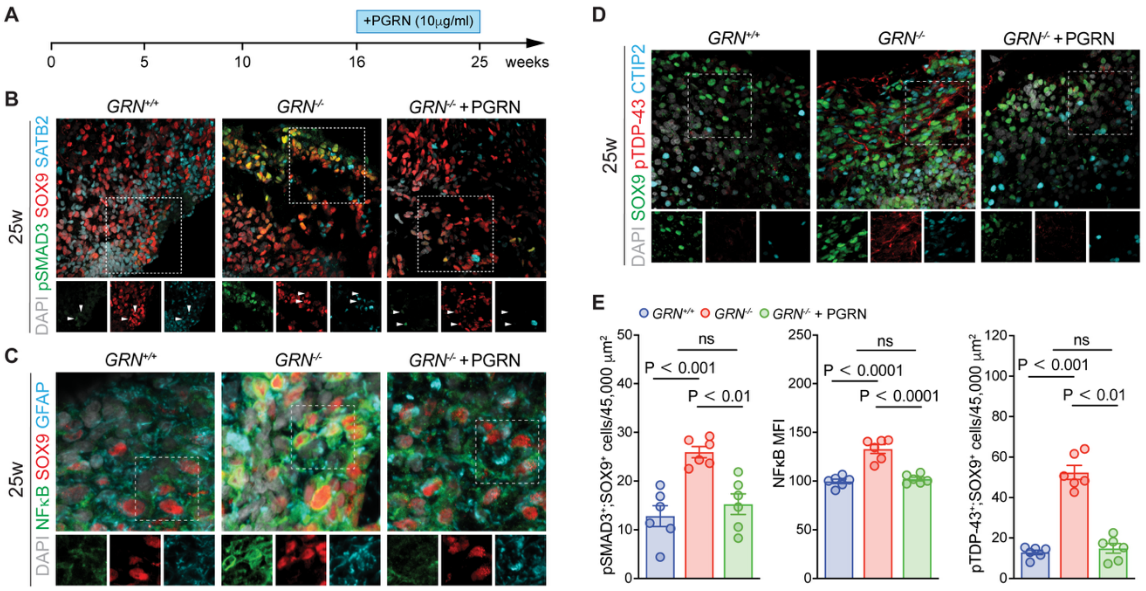

Supplementary Figure S8. Related to Figure 5. Progranulin replacement rescues TGFβ activation and pTDP-43 phenotype in *GRN*$^{-/-}$ cortical organoids. (A) Schematic diagram indicating Progranulin replacement for *GRN*$^{+/+}$ and *GRN*$^{-/-}$ cortical organoids from 16 to 25 weeks. **(B-D)** Confocal images for pSMAD3, SOX9, and SATB2 (B), NFκB, SOX9, and GFAP (C), SOX9, pTDP-43, and CTIP2 in 25 weeks *GRN*$^{+/+}$ and *GRN*$^{-/-}$ cortical organoids, and *GRN*$^{-/-}$ cortical organoids treated with recombinant human Progranulin (10 μg/ml). (E) Quantification of the number of pSMAD3^{+};SOX9^{+}, the intensity of NFκB in SOX9^{+} cells, and the number of pTDP-43^{+};SOX9^{+} cells in *GRN*$^{+/+}$ organoid, untreated *GRN*$^{-/-}$ cortical organoids, and *GRN*$^{-/-}$ cortical organoids treated with Progranulin. Data represent mean ± SEM. Statistics used two-way ANOVA with multiple comparisons.

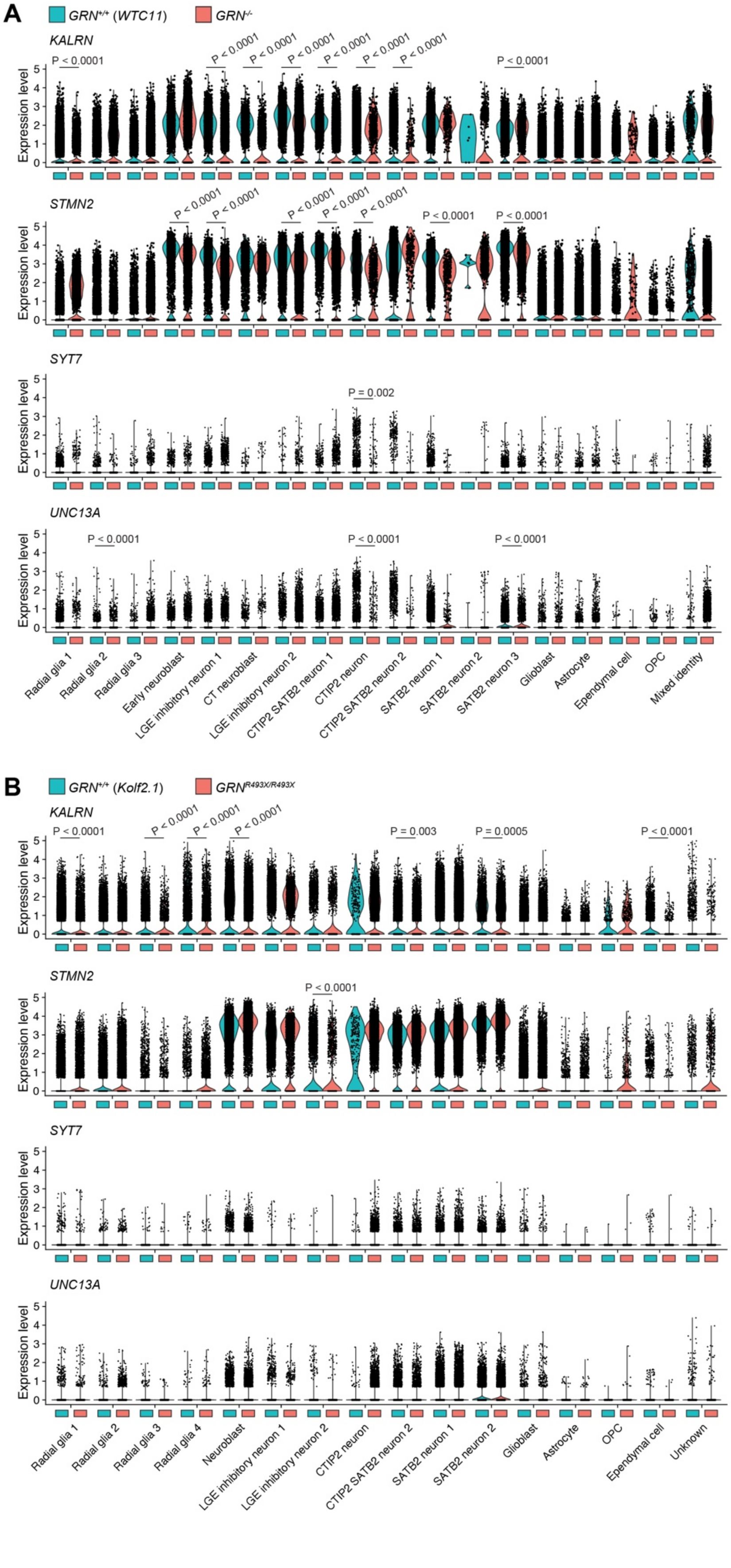

A
GRN+/+ (WTC11)
GRN-/-
KALRN
STMN2
SYT7
UNC13A
Expression level
P < 0.0001
P = 0.002
Radial glia 1
Radial glia 2
Radial glia 3
Early neuroblast
LGE inhibitory neuron 1
CT neuroblast
LGE inhibitory neuron 2
CTIP2 SATB2 neuron 1
CTIP2 neuron
CTIP2 SATB2 neuron 2
SATB2 neuron 1
SATB2 neuron 2
SATB2 neuron 3
Glioblast
Astrocyte
Ependymal cell
OPC
Mixed identity
B
GRN+/+ (Kolf2.1)
GRNR493X/R493X
KALRN
STMN2
SYT7
UNC13A
Expression level
P < 0.0001
P = 0.003
P = 0.0005
Radial glia 1
Radial glia 2
Radial glia 3
Radial glia 4
Neuroblast
LGE inhibitory neuron 1
LGE inhibitory neuron 2
CTIP2 neuron
CTIP2 SATB2 neuron 2
SATB2 neuron 1
SATB2 neuron 2
Glioblast
Astrocyte
OPC
Ependymal cell
Unknown

Supplementary Figure S9. Related to Figure 6. Expression of *KALRN*, *STMN2*, *SYT7*, and *UNC13A* in *GRN*$^{+/+}$, *GRN*$^{-/-}$, and *GRN*$^{R493X/R493X}$ cortical organoids. (A) Violin plots of *KALRN*, *STMN2*, *SYT7*, and *UNC13A* transcript levels in all cell clusters in *GRN*$^{+/+}$ and *GRN*$^{-/-}$ cortical organoids show modest but significant down-regulation of *KALRN* and *STMN2* in selective cell clusters in *GRN*$^{-/-}$ cortical organoids. However, no difference is detected in the transcript levels of *SYT7* and *UNC13A* in all cell clusters between *GRN*$^{+/+}$ and *GRN*$^{-/-}$ cortical organoids. **(B)** Similar to *GRN*$^{-/-}$ cortical organoids, *KALRN* and *STMN2* transcript levels are reduced in *GRN*$^{R493X/R493X}$ cortical organoids in selective cell clusters, whereas no no difference is detected in the transcript levels of *SYT7* and *UNC13A* in all cell clusters between *GRN*$^{+/+}$ and *GRN*$^{R493X/R493X}$ cortical organoids.

A

	5w $GRN^{R493X/R493X}$	5w $GRN^{+/+}$ (*Kolf2.1*)	16w $GRN^{R493X/R493X}$	16w $GRN^{+/+}$ (*Kolf2.1*)	25w $GRN^{R493X/R493X}$	25w $GRN^{+/+}$ (*Kolf2.1*)
Cells	44,895	45,060	30,343	34,386	41,369	46,141
Biological replicates	4	4	4	4	4	4
Experimental replicates	2	2	2	2	2	2

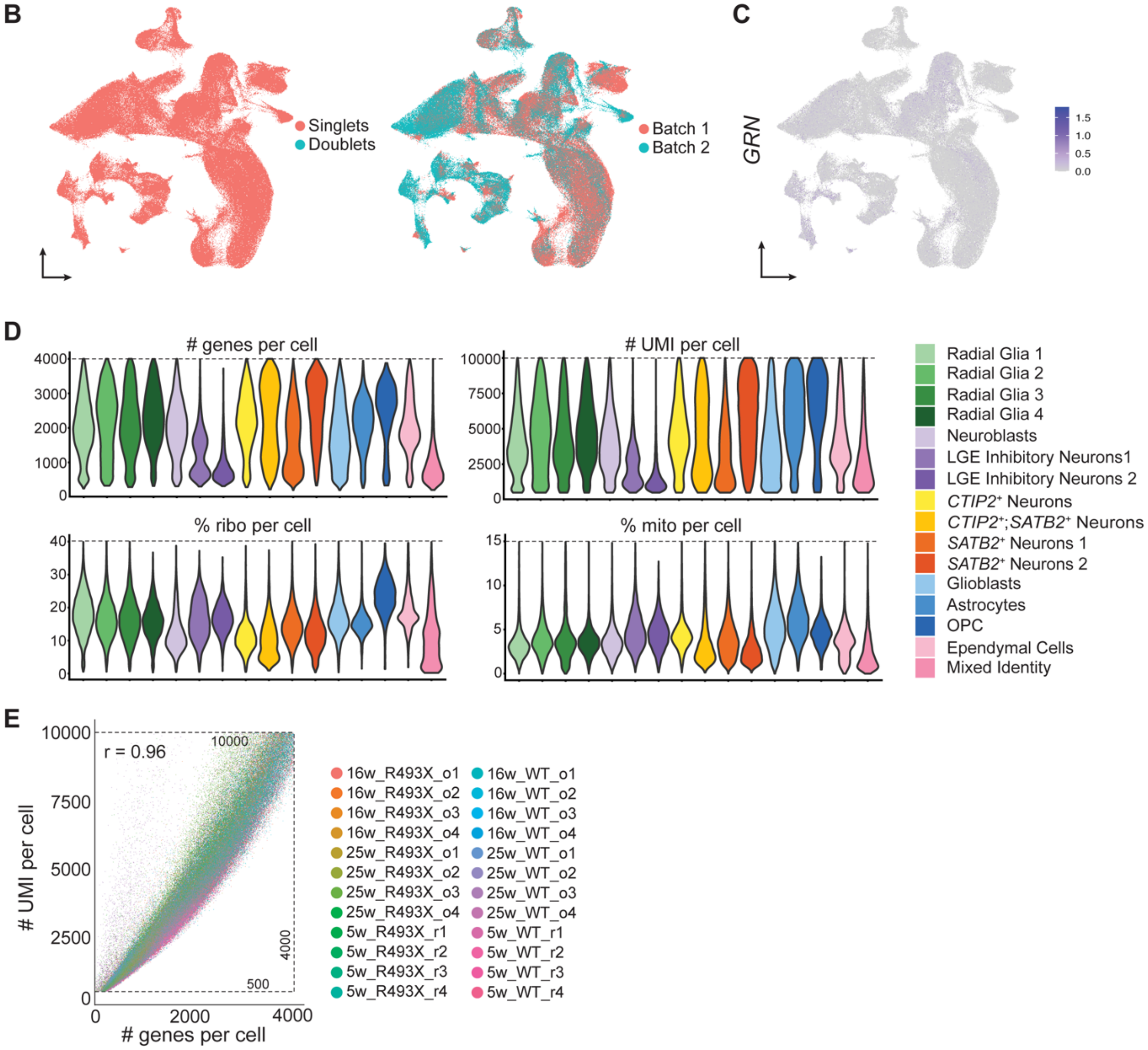

Supplementary Figure S10. Related Figure 8. Single-cell transcriptomic analyses of *GRN*$^{+/+}$ and *GRN*$^{R493X/R493X}$ cortical organoids. (A) Table showing the number of cells, biological replicates, and experimental replicates per age and genotype. **(B)** UMAPs grouped by singlet vs doublet (left) and experimental batch (right). **(C)** Feature plot showing the expression of *GRN*. **(D)** Violin plots grouped by cluster showing the number of genes per cell (top left), number of UMI per cell (top right), percentage of

ribosomal genes per cell (bottom left), and percentage of mitochondrial genes per cell (bottom right). **(E)** Scatterplot grouped by sample showing the correlation between the number of unique molecular identifiers (UMI) per cell and the number of genes per cell.

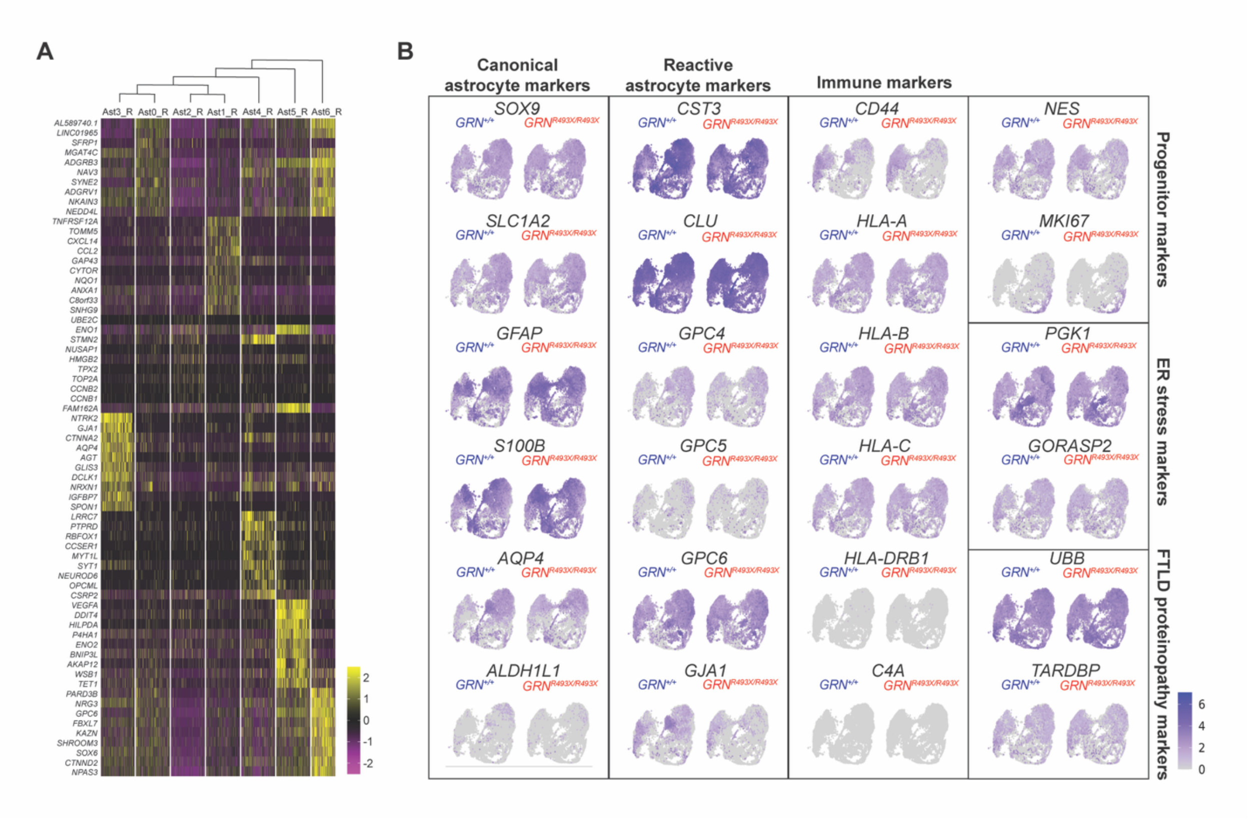

Supplementary Figure S11. Related to Figure 9. Transcriptomic profiles of astrocyte subclusters in *GRN*$^{+/+}$ and *GRN*$^{R493X/R493X}$ cortical organoids. (A) Heatmap of top DEGs in each astroglia cluster. Clusters are sorted by hierarchal similarity (top). **(B)** Feature plots of canonical astrocyte markers, reactive astrocyte markers, immune markers, progenitor markers, ER stress markers, and FTLD proteinopathy markers.
