# Supplementary figures and images for: TGF-β signaling promotes astroglial activation and TDP-43 proteinopathy in organoid models of frontotemporal lobar degeneration
Source: J Clin Invest. 2026 Jun 16;136(14):e190035. doi: 10.1172/JCI190035 (PMC13367973; doi:10.1172/JCI190035)

Figure 4L

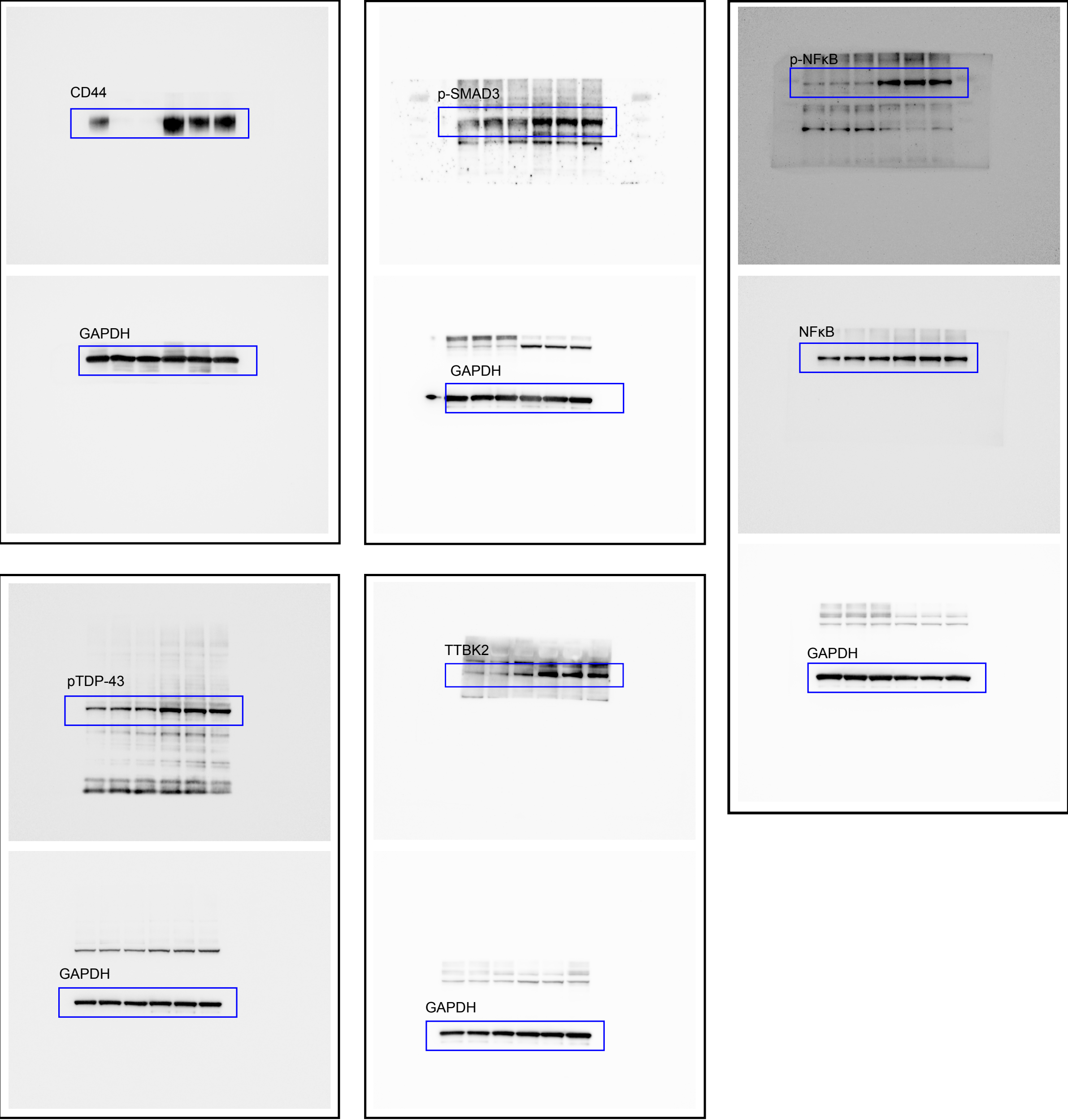

Figure 5F

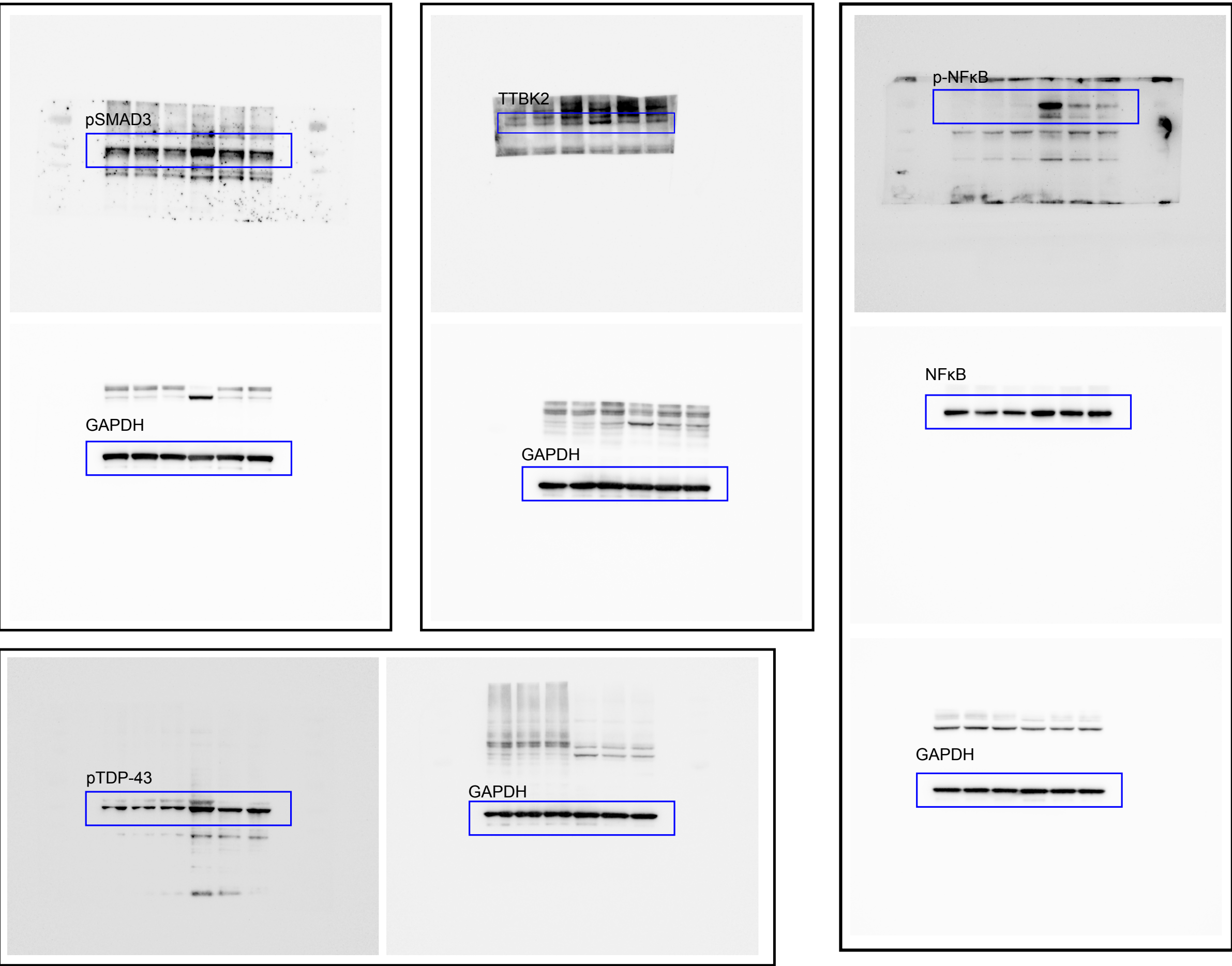

Supplement: Unedited blot and gel images [file jci-136-190035-s094.pdf]
